
JOURNAL INFORMATION
==============================
NLM Title Abbreviation: J Orthop Traumatol
Journal ID: J Orthop Traumatol
NLM Journal ID: 101090931

Journal of Orthopaedics and Traumatology : Official Journal of the Italian Society of Orthopaedics and Traumatology

ISSN: 1590-9921
EISSN: 1590-9999
Publisher: Springer

ARTICLE INFORMATION
==============================
PMCID: PMC4417748
DOI: 10.1007/s10195-011-0150-2
Article ID: 150
Article version: 1
Subjects: Events

In-Depth Oral Presentations and Oral Communications

Electronic publication date: 2011 Sep 2
Print publication date: 2011 Oct
Volume: 12
Issue: Suppl 1
First page: 89
Last page: 123
Copyright: © The Author(s) 2011
License: Open AccessThis article is distributed under the terms of the Creative Commons Attribution 2.0 International License (https://creativecommons.org/licenses/by/2.0), which permits unrestricted use, distribution, and reproduction in any medium, provided the original work is properly cited.
License URL: https://creativecommons.org/licenses/by/2.0/

Keywords: Bone Mineral Density, Total Knee Arthroplasty, UHMWPE, Oswestry Disability Index, Aseptic Loosening
issue-copyright-statement: © The Author(s) 2011

==============================
J Orthopaed Traumatol (2011) 12 (Suppl 1):S89–S123
DOI 10.1007/s10195-011-0150-2

4 OCTOBER 2011

### In-Depth Oral Presentations and Oral Communications

### IN-DEPTH ORAL PRESENTATIONS

### *AT09—TRAUMATOLOGY 1*

### 101 distal radial fractures treated with the CASLAU non bridging external fixation technique: results at an average 14-month follow-up (range, 8–20)

D. Potestio*[1], E. Theodorakis[2], F. Laurenti[2]

[1]Casa di Cura Villa del Rosario (Rome, IT);
[2]Policlinico Umberto I (Rome, IT)

**Introduction** Distal radial fractures represent 17% of fractures in the Italian E.R. At the University Hospital “Policlinico Umberto I” of Rome E.R. we have started treating these fractures with a new fixation system designed by us: the CASLAU. This system provides a non-bridging external fixation. The synthesis is guaranteed by two or more K-wires which can be intramedullary or x-crossing the cortex and/or inter-fragmentary. These K-wires are connected with two radial pins by an external bar. This radial to radial system gives stability to the fracture and allows the patient to move the wrist immediately. We remove this fixation system after 40 days.
**Materials and methods** From July 2008 to July 2010 we treated 127 distal radial fractures. Evaluation outcomes are presented only for the 101 patients that, at the present time, have a follow-up longer than 8 months. Clinical assessment was performed every 7 days until removal of external fixation system, then at 2, 3, 6 and 12 months. Radiographic assessment was performed at 30 and 40 days, consequently at 2, 3, 6 and 12 months. Outcome was measured on the basis of range of motion, grip and pinch strength, DASH and PRWE scores. A questionnaire was used to determine patient satisfaction, and a detailed analysis of complications was carried out.
**Results** All patients had excellent or good results and were satisfied with the clinical outcome. At 60 days after surgery 94% of patients showed complete clinical and functional recovery. After 3 months 100% of patients showed complete clinical and functional recovery. After 6 and 12 months no modification of the obtained result was detected.
**Discussion** Considering the immediate movement recovery and the minimal invasive system CASLAU non bridging external fixator is on our opinion a useful tool for DRF’s treatment.
**Conclusions** On our series, at an average 14-month follow-up, the CASLAU system showed good to excellent results.

### Corticocancellous iliac graft, dynamic compression plate, and injection of autologous platelet for the treatment of acquired aseptic ulna non-unions: analysis of 10 cases

L. Tarallo*[1], R. Mugnai[1], R. Adani[2], F. Catani[1]

[1]Clinica Ortopedica e Traumatologica, Università degli Studi di Modena e Reggio Emilia (Modena, IT);
[2]U.O.C. di Chirurgia della Mano e Microchirurgia, Policlinico G.B. Rossi (Verona, IT)

**Introduction** The aim of this work is to evaluate the results obtained after the treatment of non-union ulna shaft with bone defect between 3 and 4 cm using L.C.P. plate 3.5 mm with locking screws in association with corticocancellous iliac graft and injection of autologous platelet gel.
**Materials and methods** In a period between March 2004 and March 2007, 10 patients were treated (6 man, 4 women) for non-union ulna shaft, using L.C.P plate and autologous bone graft. The bone defects ranged between 1 and 4 cm with an average of 2.2 cm. The time between initial trauma and surgery ranged from 7 to 15 months.
**Results** The healing of the nonunion was observed in 8 of 10 patients, at a median time of 5 months after surgery. Longer recovery times were observed in cases of nonunion treated with cortocospongiosous graft. The functional results were classified using the system described by Anderson that provided 7 excellent results (70%), 1 good result and 2 failures for the non-union occurred 6 months after surgery.
**Conclusions** Non-union of the ulna is most commonly observed when the fracture reduction has been inadequate or where the means of synthesis has not provided the necessary stability. The larger part of the nonunion healed on average 4.5 months, the functional result was excellent in 70% of cases. The forearm skeletal shortening cannot be tolerated without undergoing a radical upheaval in the function, the fixation and shortening is always to be avoided. The best results were obtained in cases where the bone gap was smaller, resulting in an almost total functional restoration.

### Management of complex-exposed leg fractures and use of vacuum-assisted closure (VAC) therapy

R. Politano*, A. Longhi, A. Di Giulio, D. Morici

Azienda Ospedali Riuniti (Ancona, IT)

**Introduction** The treatment of exposed fractures is a surgical urgency, and bone damage often associates with neurovascular or soft tissues lesions. High energy multiple-trauma patients often suffer from complex-exposed fractures.

**Materials and methods** Two-hundred eighteen leg fractures have been treated in our Institution from January 2006 to December 2010 (64 female/164 male; median age, 47 years [range17–86]) of which 132 diaphyseal tibial fractures, 32 exposed fractures, and 5 amputations.

**Results** The management of complex-exposed tibial fractures critically requires strict collaboration and joint decisions between orthopaedic surgeon, vascular surgeon and plastic surgeon. Treatment must begin in the emergency room giving wound contamination for granted and considering the need for immediate therapy and damage control. External fixation allows a stable fixation, not only in limb fractures type III (according to Gustilo-Anderson) but also in severe multiple-trauma patients, providing a better patient care in the resuscitation department and an effective damage control, planing for a second-look for wound contamination.

**Discussion** The acute treatment has to allow an effective fracture stabilization, the resection of all the necrotized tissue and the preservation of bone and soft tissue vascularization. It may involve a plan for a second-look with vacuum-assisted closure (VAC) therapy.

**Conclusions** In our experience VAC therapy is achieving a prominent role in the treatment of complex-exposed leg fractures, decreasing both patient complications and costs.

### Two-step treatment of distal tibial pilon fractures: early stabilisation

W. Daghino*, T. Benigno, E. Albenga, B. Battiston, G. Vasario

Ospedale CTO (Turin, IT)

**Introduction** Soft-tissues involvement in distal tibial pilon fractures is a major determinant in outcome. These lesions can be optimally managed by a two-step treatment protocol: in the acute phases, an early temporary stabilisation with a spanning external fixator can allow, in the following 5–14 days, a careful monitoring of the soft-tissues condition before definitive ostheosynthesis.

**Materials and methods** A tripolar configuration of the early external fixator is showed. This frame configuration allows to minimise the surgery-related soft tissue trauma. Surgical technique and implant characteristics are explained in detail. From January to December 2010, 10 patients were treated with this protocol at the CTO-Hospital in Turin, Italy.

**Results** All external fixators were placed without the need of intraoperative fluoroscopy. In 5 patients early fixation was performed under simple sedation, while in 5 patients a spinal analgesia was administered. Mean procedure time was 10 min. CT scan was performed in all cases, after ligamentotaxis through distraction and fixation, to assess reduction and fracture characteristics. No implants required removal before definitive surgery; no intraoperative complications were registered in frame positioning. No implant-related complications were recorded during final surgery. Definitive ostheosynthesis was performed by plate and screws in 8 cases. In 2 cases a circular external fixator, associated with percutaneous reduction and screws placement, was adopted.

**Discussion** Two-step treatment of tibial pilon fractures allows, by the means of specifically designed frame configurations, to minimise the surgical impact. With the tripolar configuration external fixation was performed in all cases in shock room, without the need of intraoperative fluoroscopy. No general or spinal analgesia is required. After stabilisation, pain management, nursing and patient mobility are optimized. Fracture stabilisation and re-alignment through distraction allow optimal soft-tissue management, accurate assessment of fracture and thorough pre-operative planning.

**Conclusions** Tri-polar frame construction could represent an effective option for early treatment of tibial pilon fractures, reducing surgery-related trauma and complications and allowing an optimal soft-tissues care.

### Immediate surgery advantages in ankle fractures-dislocations

D. Bazzoni*, A. Mazzucco, V. Occhipinti, M. Gadaleta, L. Trentani, M. Merlo

Ospedale di Circolo di Busto Arsizio (Busto Arsizio-Varese, IT)

**Introduction** Bimalleolar or trimalleolar fractures associated with dislocation of the ankle represent a frequent lesion in lower limbs traumatology. The presence, at the same time, of bones, ligaments and soft tissues lesions, classifies them as complex injuries. To avoid soft-tissues complications surgery must be performed without swelling: this condition is present until 6–8 h from injury and some days after provisional reduction. As concerning long-term results of ankle function, many authors report no statistical difference if considering timing of definitive surgery.

**Materials and methods** We reviewed all ankle bi or trimalleolar fractures with dislocation, close and open, treated in our Unit in the last 5 years. We treated 42 lesions; the first choice was always early treatment; delayed treatment (after resolution of swelling) was done in presence of contraindications or logistic problems for early treatment. Immediate surgery was performed in 33 cases, delayed surgery in 9 cases. Almost all early treatments were ORIF, even if in presence of Gustilo type 1 open fractures although with good soft tissues conditions.

**Results** We considered for each patient long-term ankle function, numbers of operations, hospital stay, time of wound healing and complications. The patients who received early treatment had better outcomes. We present our results.

**Discussion** Many authors report series of patients suffering ankle fractures-dislocations. Considering only long-term ankle function, there is no difference between patients who had early or delayed treatment. We think that it is more precise to evaluate these patients also with other parameters such as number of operations, duration of hospital stay, time of wound healing and complications.

**Conclusions** In our experience, early treatment of bi and trimalleolar ankle fractures associated with dislocation is preferable; ORIF, if possible, is an all-in-one technique and there is no need of further operations.

**Suggested reading**

1. Høiness P, Strømsøe K (2000) The influence of the timing of surgery on soft tissue complications and hospital stay. A review of 84 closed ankle fractures. Ann Chir Gynaecol 89(1):6–9

### Tibial pilon fractures: results in the medium- to long-term

T. Binda*, M. Faluomi, F. D'Angelo, P. Cherubino

Clinica Ortopedica, Università dell'Insubria (Varese, IT)

**Introduction** Tibial plafond fractures are generally caused by high-energy trauma and are often associated with soft tissues compromission. The aim of this study is to evaluate the clinical and radiographic results in the medium and long-term of surgical treatment in tibial pilon fractures, considering internal synthesis techniques and external fixation.
**Materials and methods** In the period between January 2000 and September 2007, 47 patients were treated, in 9 cases (19.1%) fractures were exposed. 18 patients (38.2%) underwent internal synthesis, 29 (61.7%) external fixation. The evaluation of the results was conducted according to the clinical criteria proposed by Ovadia and Beals, with the addition of a subjective evaluation form SF-36 and the radiographic Kellgren-Lawrence Grading Scale.
**Results** At a follow-up period of 79 months (range: 39–132) an excellent objective result was observed in 58.62% of cases of fractures treated with external fixation and 83.33% of cases treated with internal synthesis; good results in 24.13% of the first group and in 11% of cases in the second group. Moderate or insufficient results were detected in 17, 25% of cases treated with external synthesis and 5, 56% of patients treated by internal fixation.
**Discussion** The good results obtained suggest that a multidisciplinary approach, with the help of plastic surgeon, a careful preoperative planning, a little traumatic surgery aimed at anatomic reduction of articular fragments and their stable fixation represent the best strategy for the treatment of these fractures. The main prognostic factor is the severity of initial injury in terms of type of fracture of compromised soft tissues. According to our experience, we believe that the easier lesions, without compromising the soft tissues, could be treated with internal fixation. In case of more serious skeletal injuries the aim of the surgeon should be the research of an anatomic reduction as much as possible, understanding that the fracture's personality affects the clinical outcome. The development of osteoarthritis at distance has been shown to be directly related to the type of initial skeletal lesion.

### *AT10—TRAUMATOLOGY 2*

### Dynamic internal fixator

C. Battiato*

Azienda ASUR 13 (Ascoli Piceno, IT)

**Introduction** We herewith show the results obtained in diaphyseal as well as distal and proximal epiphyso-metaphyseal femoral fracture treatment by means of the DIF innovative system. Such system belongs to the so-called "internal fixators" category, i.e. angular stability plates which have been modified so as to become dynamic, when required. Within the action limit of the external fixators, this system, therefore, allows modulating the need to increase compression. The biomechanics osteo-implant system theory is discussed and the clinical and radiographic results obtained in fractures and pseudoarthrosis treatment are shown.
**Materials and methods** The DIF was applied to 10 patients with different femoral fracture with patterns at risk of pseudoarthrosis. In all these patients the system was dynamized at a period between the 40th and the 60th day after the implantation. X-rays were taken every 30 days. The follow-up was for all the 10 patients carried on until the radiographic evidence of bone repair.
**Results** In all patients we observed a fast bone recovery and no case of implant failure.
**Discussion** We think that the possibility of a plate-sliding system discharges the angular flexion pressure (flexing movement) becoming a sliding movement and thus an axial compression.
**Conclusions** We think that DIF allows the possibility of increasing fracture site compression when charging during the healing process. Furthermore, the possibility of a plate-sliding system discharges the angular flexion pressure (flexing movement) becoming a sliding movement and thus an axial compression.

### Comparison between two different intramedullary nails in the treatment of intertrochanteric fractures

C. D'Arrigo*, A. Carcangiu, D. Perugia, A. Speranza, R. Alonzo, S. De Sanctis

Azienda Ospedaliera S. Andrea, Università di Roma "Sapienza", II Facoltà di Medicina e Chirurgia (Rome, IT)

**Introduction** The objective of the study is to compare 1 year results in intertrochanteric fractures treated with TGN (trochanteric Gamma Nail) and PFNA (Proximal Femoral Nail Antirotation).
**Materials and methods** 97 out of 110 consecutive patients treated for intertrochanteric fracture of femur were included in this study. Thirteen of these were lost at the first follow-up and excluded from the study. All fractures were closed, isolated, unstable intertrochanteric fractures of famous, type 31-A1, 2, 3 according to AO Classification. Clinical and radiological follow-up were available. Surgical time, blood loss and complications have been considered.
**Results** The mean operative time for the TGN group was significantly higher than in the PFNA group (62 and 45 min respectively) with a $p = 0.04$. The mean blood loss was significantly higher in the TGN group (285 ml; SD 145) in relation to PFNA group (226 ml; SD 136) with a $p = 0.03$. Also rate of complications were higher in the TGN group ($p = 0.01$). We also noted that all but one case of complications in the group treated with TGN have occurred in patients older than 80aa. Clinical outcomes were good for both groups.
**Discussion** In recent years, due to the aging population, the incidence of extracapsular fractures is gradually increased. Treatment of intertrochanteric fractures using both the TGN and PFNA implants is quite reliable. Intra-operative and post-operative complications in the TGN group were associated with a longer operative time and a higher blood loss. Moreover all but one of the procedure related complications were observed in very elderly patient.
**Conclusions** The choice of intramedullary system should be made taking into account the type of fracture, bone quality and patient characteristics. Our results show that the use of PFNA for treatment of intertrochanteric fractures should be recommended in case of elderly and osteoporotic patients, while TGN should be used in more severely displaced fractures and in patients with a slight better bone mineral density.

### Intramedullary nailing's complications of the proximal femur fractures

P. Barca*, R. Pascarella, A. Maresca, G. Melucci, L. Prosperi

Ospedale Maggiore (Bologna, IT)

**Introduction** The intramedullary nailing is a technique that was born and grew thanks to the contribution of military surgeons who have over time been trying to solve as soon as possible disability of the military in war, although it is hard to determine the first surgeon who performed an operation of intramedullary nailing. Among the first surgeons using this technique, certainly there are Roux (August 1833), Cloquet, Dupuytren, Cruvelhier. During the World War I, a true pioneer in the study of intramedullary fixation of femoral shaft was the British military surgeon E.W. Hey Groves. In 1925 Smith Petersen introduced the flanged nail and this was a revolution compared to the previous nails. In 1932, the Swede Sven Johansson proposed his cannulated nail positioned on a guide wire represented by a Kirscnher, leading from an intra-articular method to the extra-articular. But it was during the World War II that a German military surgeon, Gerhard Küntscher, revolutionized the treatment of fractures by intramedullary nailing. The first intervention of the femoral diaphyseal nailing was performed in November 1939. Since then different types of intramedullary nails were designed up to the first nail in the range 1980 through Grosse, arriving finally to the modern Gamma 3 in 2003.
**Materials and methods** From 01/01/1997 to 31/12/2010 for the treatment of lateral fractures of the proximal femur were implanted at the Hospital Maggiore of Bologna Standard Range 525 nails, 422 and 1099 nails Short Gamma 3. In all cases we used the standard technique, using the dedicated instruments.
**Discussion** Among the complications associated with the use of nails in the series we can distinguish between intra-and postoperative. Among the first ones we should include the intraoperative fracture, poor reduction, malposition of the distal locking screws, the screw cephalic articulation and finally breaking the olive of the guide wire. The postoperative complications are most often haematomas, consolidation of the fracture in varus, the cut outs, delayed union, nonunion, necrosis, cephalic and post-operative diaphyseal fractures.
**Conclusions** For each of the above complications related to the use of nail range there are many steps that can be taken either at the time surgery or post-operatively in order to avoid them. In conclusion we can say that most complications are not related to the means of synthesis, but to the operator.

### Treatment of atrophic pseudoarthrosis of the distal femur with LRS single-axis external fixator

G. Manetti, L. De Luca*, M. Sangiovanni, M. Valori

SOD Ortopedia Traumatologica, A.O.U. Careggi (Florence, IT)

**Introduction** Nonunion of the distal femur fracture concerns the 6% of cases, whose treatment can vary.
**Materials and methods** At our Department were treated 4 cases of atrophic pseudoarthrosis of the distal femur between 2007 and 2009. The patient was positioned with the affected limb in traction. Using a lateral surgical approach to distal femur, a bone resection and debridment of the point of nonunion and its immediate compression were performed. In 3 cases we used a transplant with OP-1. It is positioned a single-axis external fixator (Limb Reconstruction System (LRS)) and the osteotomy was performed at the proximal femur. From the day after the patient began CPM. After 8 days the proximal osteotomy distraction was started (0.25 mm every 6 h). The patient walked with load as tolerated and two crutches. Was performed a clinical and X-rays follow-up every 20 days for the first 2 months and then every 40 days.
**Results** All patients were reviewed. Sites of compression and distraction were all consolidated without deformation of the regenerate. The average lengthening was 5.5 cm (range 5–7), the average time of the fixator was 238 days (range 225–248), 41 days per cm of distraction. The average residual limb-length discrepancy was 1.5 cm (range 0.9–2). We achieved an average ROM of the knee of 95° (range 80°–120°). We recorded two cases of superficial infections of the fiches and no deep infection.
**Discussion** The treatment of atrophic nonunion requires the removal of necrotic and fibrous tissue for research in a viability bone resulting in a bone gap. Often the treatment of this gap requires a bone graft. The graft must restore the stability of the fracture site and make a biological stimulus that increases the blood supply. With the method described we obtained an immediate and viability contact, reducing the amount of bone graft, a good stability and a gradual recovery of the asymmetry. The disadvantages of this method are the stiffness of the knee, frequent dressings to prevent infections, especially superficial. In addition, patient compliance should be good to put up the bulk of the external fixator and the long treatment times.
**Conclusions** We believe this system a good alternative for the treatment of nonunion of the distal femur with bone gap.

### *AT11—SPINE*

### Decision making algorithm on posterior osteotomy type to be performed in adult spinal deformity: role of MRI in determining flexibility

G. Bakaloudis*, M. Bochicchio, S. Astolfi

U.O.C di Chirurgia Vertebrale, Ospedale San Carlo di Nancy, IDI (Rome, IT)

**Introduction** Purpose of the present study was to assess the reliability of a treatment based algorithm on posterior osteotomy type to be performed in adult spinal deformities as assessed by preoperative flexibility on MRI.
**Materials and methods** A consecutive series of 49 adult patients (range, 60–82 years) surgically treated by means of an all posterior, pedicle screws based arthrodesis was reviewed. In 33 patients a degenerative scoliosis was present, in 7 a "fixed sagittal imbalance", in 5 a "flat back syndrome", and in 4 a sharp, angular kyphosis. In 12 cases a m-Ponte procedure was performed, in 5 a PSO, in 4 a pVCR. The reliability of the algorithm used was determined considering as "successful" a procedure that obtained a main deformity correction >50%, a global coronal/sagittal imbalance <2 cm, a final lordosis >45°. Contingency table analysis, Pearson correlation coefficient and univariate analysis of variance were used.
**Results** At a minimum 12-month follow-up (range, 12–22) all patients were found well balanced both in the coronal and sagittal plane, with a main curve correction up to 65% (range, 53–72; $p < 0.001$). A strong statistical ability of the algorithm was observed in predicting a "successful" procedure (sensitivity = 0.913, PPV = 0.874, NPV = 0.865), with both coronal and sagittal plane flexibility, as assessed on MRI, been highly correlated (r = 0.803, $p < 0.001$) with final outcome. Final SRS-30 and ODI assessment was 4.6 and 12.8 respectively, with all patients well satisfied. No permanent neurologic or any other implant related complications have been reported.

**Discussion** Posterior osteotomies have enhanced the ability to treat severe, complex spinal deformities. Recently, Bridwell et al. have proposed a flow chart describing a decision making process on type of posterior osteotomy to perform in adult spinal deformity. Nevertheless, the role of flexibility (preoperative-flexibility/preoperative) as calculated on MRI has never been reported.

**Conclusions** According to present series, the algorithm used has been highly predictive in decision making on type of posterior osteotomy to perform in adult spinal deformity (sensitivity = 0.913). The decision whether to perform a wide release vs. Ponte vs. PSO vs. PVCR remains crucial for the overall outcome of such demanding procedures. The use of coronal and sagittal MRI in determining flexibility can obviate the need for further radiation based pre-operative imaging studies.

### Surgical treatment of flat back

A. Cioni*, K. Martikos, M. Di Silvestre, F. Lolli, S. Giacomini, F. Vommaro, E, Maredi, T. Greggi

Chirurgia delle Deformità del Rachide, IOR (Bologna, IT)

**Introduction** Loss of physiologic lumbar lordosis (flat back) represents a pathologic condition, secondary to degenerative processes or iatrogenic causes, and consists in the forward flexion of the body with difficulty during ambulation, lumbar pain, neurological symptoms and limitation off normal life. Osteotomy according to pedicle subtraction or Smith Pettersen technique can be performed for the correction of the sagittal plane deformity.

**Materials and methods** Retrospective evaluation of 80 consecutive patients surgically treated for flat back, by posterior instrumentation and associated pedicle subtraction or Smith Pettersen osteotomy. 35 patients presented an associated deformity in the coronal plane. Pedicle subtraction osteotomy was performed in 39 cases and Smith Pettersen osteotomy was performed in 41 cases. Patients' men age was 55 years (min 31–max 70). All surgical procedures were performed from 1998 to 2010. We analyze the 2 surgical techniques and compare clinical and radiographic result, gathered after a mean 25 months follow-up. We used SF-36, ODI, VAS questionnaires for the evaluation of clinical outcome and radiographic data related to sagittal balance obtained upon plane X-rays.

**Results** In both groups sagittal plane correction of deformity was significant. Mean sagittal plane correction was greater in the group treated by pedicle subtraction osteotomy. In both groups clinical results showed a significant improvement of life quality. Early complications rate was 47% in the pedicle subtraction osteotomy group and 25% in the Smith Pettersen osteotomy group ($p < 0.05$), while the loss of correction was 23% e 32% respectively ($p < 0.05$).

**Conclusions** Smith Pettersen and pedicle subtraction osteotomy are valid surgical techniques for the treatment of flat back, able to improve patient's life quality. Pedicle subtraction osteotomy allows greater correction, but represents a surgical procedure that requires excellent skill, with an associated high rate of potential complications.

### A new device for the protection of the dura during en bloc resection

A. Gasbarrini*, R. Ghermandi, S. Bandiera, G. Barbanti Bròdano, S. Terzi, L. Boriani, A. Corghi, S. Colangeli, C. Simoes, L. Babbi, F. Santoro, S. Boriani

Istituto Ortopedico Rizzoli (Bologna, IT)

**Introduction** En bloc resection is indicated in selected cases of primary spine tumors and spine metastasis. In the last years special devices were developed in order to help the surgeons to perform the procedure and to decrease morbidity and complications. The aim of this study is to compare retrospectively the posterior resection with traditional osteotomy versus the use of a special device (PROMID) safely connected to one of the rod to facilitate a complete osteotomy by Gigli saw and fully protect the dura until the removal of the specimen.

**Materials and methods** A total number of 25 patients underwent posterior resection.

*Group 1*: 11 patients (4 male, 7 female), average age 53 years (range 24–67 years), affected by spine tumor (9 metastasis, 2 primary) treated with the use of PROMID (10 cases thoracic localisation; 1 case lumbar localisation).

*Group 2*: 14 patients (4 male, 10 female), average age 48 years (range 26–83 years), affected by spine tumor (8 metastasis, 6 primary) treated with traditional osteotomy (13 cases thoracic localisation; 1 case lumbar localisation).

Surgical time, pre and post operative haemoglobin values, complications, and transfusions required were recordered.

**Results** Mean follow-up was 13 months (min 2.4–max 36.6) for group 1, 45 months (min 3.7–max 112) for group 2. Mean surgical time was 294 min (min 255–max 360) for group 1, 388 min (min 270–max 510) for group 2. The mean preoperative haemoglobin value was the same for the two groups: 12.5; the mean post-operative value after 24 h was 11.8 for group 1 and group 2. The mean blood volume transfused was of 2200 ml (35.6 ml/kg; min 1,200–max 5,600) for group 1, 3,900 ml (53 ml/kg; min 2100–max 8100) for group 2. No major complications were recordered. Two minor complications occurred.

**Discussion** Despite the high rate of morbidity and complications, en bloc resection represents the treatment of choice in selected cases of spine tumors (primary or metastasis). The development of specific special devices and new surgical techniques are very important steps in order to improve the final outcome.

**Conclusions** In this particular kind of surgery, the final result is strictly connected to the patient selection and to the surgical team experience. New specific devices like PROMID are useful to decrease the impact of complications and morbidity related to this surgical procedure.

### Surgical treatment of evolutive early onset scoliosis: characteristics and indications of modern surgical techniques

T. Greggi*, F. Lolli, F. Vommaro, K. Martikos, E. Maredi, M. Di Silvestre, S. Giacomini, A. Cioni, M. Spina

Istituto Ortopedico Rizzoli (Bologna, IT)

**Introduction** A rapidly progressive scoliosis of childhood determines, in a very short time, a severe deformity of the spine and rib cage, resulting in impaired lung function. An early fusion may lead to a decreased size of chest and lungs, worsening the life expectancy. Growth sparing techniques, based on growing and VEPTR-like instrumentations, represent an innovation, allowing to check the evolution of the deformity (spinal and thoracic) without compromising the growth of the column. There may be, by spine surgeons, doubts, due to the fact that these techniques expose patients to a large number of interventions, necessary to lengthen the instrumentation, and for the large number of mechanical complications described in the literature. Our work is aimed at evaluating the effectiveness of "extensible" instrumentations, focusing on the specific indications of any system and the peculiarities of each technique.

**Materials and methods** In our Division we use the most modern extensible systems since 2006. 19 patients, 9 males and 10 females, with a mean age of 6.8 years (min 2–max 11) were included in the study. All patients were affected by scoliosis, idiopathic in 7 cases, syndromic in 6, congenital in 5 and associated with congenital heart disease in 1. In 9 cases a growing-rod was implanted, in 10 cases a VEPTR-like system. All surgeries were performed under neurophysiologic monitoring (SSEP, MEP).

**Results** At a mean follow-up of 38 months (range, 6–55) 31 new surgeries were performed, 13 in patients treated with growing rod system and 18 in those treated with VEPTR-like system. In patients treated with growing rod, 8 instrumentation lengthening and 5 revisions due to mechanical complications (rod breakage in 1 case, proximal anchors mobilization in 4 cases, made by screws in 3 cases and hooks in 1 case) were performed. In patients treated with VEPTR-like system, 10 lengthening, 4 revisions, 3 conversions and 1 instrumentation removal were performed. No case of neurological complications occurred.

**Discussion** In our experience, we can only confirm the efficacy (optimal control of the evolution of the deformity) and security (no neurological complication) of growing rod and VEPTR-like systems. The number of reoperations, however, was high (31). This experience leads us to emphasize that these techniques should be performed only in highly specialized centers and require a careful preoperative planning.

### Direct repair of symptomatic spondylolysis in young athletes: indications, surgical technique and results

F. Trucchi*[1], G. Collo[2], A. Maiello[3], E. Gennaro[3], F. Castoldi[3]

[1]Ospedale Mauriziano Umberto I (Turin, IT);
[2]Azienda Ospedaliera CTO/Maria Adelaide (Turin, IT);
[3] Università degli Studi (Turin, IT)

**Introduction** Micro-traumatic spondylolysis is one the most frequent causes of mechanical back pain in adolescent, especially in the young athlete. The purpose of this study is to describe a variation in Morsher's surgical technique of direct repair with peduncolar screws and sublaminar hooks. Moreover we describe our initial results.

**Materials and methods** In our retrospective study we selected patients on these inclusion criteria: (1) bilateral micro-traumatic spondylolysis without spondylolisthesis (according to Marchetti-Bartolozzi classification); (2) failure of conservative treatment for at least 6 months; (3) no degenerative changes in intervertebral disk; (4) back pain without radicular symptoms; (5) age between 12 and 19 years old; (6) surgical intervention between January 2003 and March 2010; (7) patients with a complete pre-surgical imaging. Our surgical technique required one single midline incision saving supraspinous ligament, removal of spondylolithic fibrous soft tissue, decortication of the defect and homologous bone grafting. Then we used a construct of 2 peduncolar screws, 2 titanium rods and 2 sublaminar hooks. We measured the clinical outcome with Oswestry Disability Index (ODI) before and 6 months after surgical treatment. Moreover at 6 months follow-up CT scans were obtained to evaluate the reconstruction of the pars interarticularis.

**Results** Eight patients (6 males and 2 females with a mean age of 18 years) underwent surgical direct repair with our technique. Mean surgical time was 130 min with not significant blood loss. We didn't observe surgical complications. Mean hospital stay was 2.5 days. Postoperative ODI varied from 4.44 to 8.8% at 6-month follow-up with a mean improvement of 29.8%. CT scans documented healing of pars interarticularis in all patients.

**Conclusions** Our technique for direct repair in micro-traumatic spondylolysis in young athletes after failure of conservative treatment showed good reliability and reproducibility. Moreover this technique does not require particular instrumentation or an important learning curve.

### Complications in mini-invasive percutaneous fixation of thoraco-lumbar fractures

M. Cappuccio*, S. Paderni, G. Scimeca, L. Mirabile, G. Bosco, F. De Iure

Ortopedia e Traumatologia, Chirurgia del Rachide, Ospedale Maggiore (Bologna, IT)

**Introduction** Mini-invasive percutaneous fixation of amielic thoracic and lumbar fractures is a valid option vs. conservative treatment and can be the first choice for politraumatic patients. We critically analyse complication rates and limits of the technique vs. conservative or open arthrodesis.

**Materials and methods** Between May 2005 and May 2010, 133 vertebral thoracic and lumbar fractures were treated (total, 101 patients): 67 were male and 34 were female; mean age 47-year-old (min. 15–max. 82); 84 patients reported only spine fractures from trauma, 17 were politrauma with average Injury Severity Score (ISS) 25,2 (min. 17–max. 34). Thoracic-lumbar passage was the main location (T12–L1). All fractures were classified according the Magerls AO classification system: mostly type A (A1 e A3) fractures, type B and C were seldom treated with a percutaneous fixation. The main surgical procedure was a monosegmental percutaneous fixation (one level above and one below the fractured vertebral body); these procedures were performed in 79 cases. Plurisegmental fixation was achieved in 22 cases. Globally 462 pedicle screws were implanted.

**Results** Complications are separately analysed: (1) *time of turning up*: intraoperative and postoperative; postoperative one can be early (in the first month after surgical procedure) or late (after the first months from the surgical procedure); (2) *gravity*: major and minor; major complications were those leading to a prolonged hospitalisation (also due to new surgical procedure) or prolonged recovery times. We reported 13 complications (13%): 5 intraoperative (5%), 6 early postoperative (6%) e 2 late postoperative (2%); 5 were minor complications (5%) and 8 major complications (8%).

**Discussion** Percutaneous mini-invasive vertebral fixation is safe with a low rate of complications. Nevertheless, any complication leading to a prolonged recovery period is considered severe since a main goal of mini-invasive fixation procedure is to quicken ambulatory and return to work.
**Conclusions** The right patient selection and surgical indication is critical. In particular a throughout information to the patient about complications and the need of surgery to remove the hardness is crucial.

### *AT12—ARTHROSCOPY AND SPORT-RELATED TRAUMAS*

### Medial collagen meniscus implant versus partial medial meniscectomy: outcomes at a minimum 10-year follow-up

G.M. Marcheggiani Muccioli*, S. Zaffagnini, T. Bonanzinga, M.P. Neri, A. Grassi, M. Nitri, A. Bondi, G. Ravazzolo, M. Marcacci

Istituto Ortopedico Rizzoli (Bologna, IT)

**Introduction** Loss of meniscal tissue can be responsible for increased pain and decreased function. The hypothesis of this study is to prove that at a minimum 10-year follow-up, patients receiving a medial collagen meniscus implant (MCMI) would present with better clinical, radiological, and magnetic resonance imaging (MRI) outcomes when compared with patients treated with partial medial meniscectomy (PMM). Cohort study, Level of evidence 2.
**Materials and methods** Thirty-three nonconsecutive patients (men; mean age, 40 years) with meniscal injuries were enrolled in the study to receive MCMI or to serve as a control patient treated with PMM. The choice of treatment was made by the patient. All patients were clinically evaluated at time 0 and at 5 years and a minimum of 10 years after surgery (mean follow-up, 133 months) by Lysholm, visual analog scale (VAS) for pain, objective International Knee Documentation Committee (IKDC) knee form, and Tegner activity level scores. The SF-36 score was performed preoperatively and at final follow-up. Bilateral weightbearing radiographs were completed before the index surgery and at final follow-up. Minimum 10-year follow-up MRI images were compared with preoperative MRI images by means of the Yulish score. The Genovese score was also used to evaluate MCMI MRI survivorship.
**Results** The MCMI group, compared with the PMM one, showed significantly lower VAS for pain ($p = 0.004$) and higher objective IKDC ($p = 0.0001$), Teger index ($p = 0.026$), and SF-36 ($p = 0.026$ for Physical Health Index; $p = 0.004$ for Mental Health Index) scores. Radiographic evaluation showed significantly less medial joint space narrowing in the MCMI group than in the PMM group ($p = 0.0003$). No significant differences between groups were reported regarding Lysholm ($p = 0.062$) and Yulish ($p = 0.122$) scores. Genovese score remained constant between 5 and 10 years after surgery ($p = 0.5$). The MRI evaluation of the MCMI patients revealed 11 cases of myxoid degeneration signal: 4 had a normal signal with reduced size, and 2 had no recognizable implant.
**Discussion** Pain, activity level, and radiological outcomes are significantly improved with use of the MCMI at a minimum 10-year follow-up compared with PMM alone. Randomized controlled trials on a larger population are necessary to confirm MCMI benefits at long term.
**Conclusions** The findings of this study support the use of meniscal scaffold to treat partial lesion of medial meniscus. Randomized controlled trials on a larger population are necessary to confirm MCMI benefits at long term.

### A new technique for arthroscopic meniscal allograft transplantation

S. Zaffagnini*, G.M. Marcheggiani Muccioli, T. Bonanzinga, A. Grassi, M.P. Neri, F. Iacono, A. Bondi, C. Musiani, M. Marcacci

Istituto Ortopedico Rizzoli (Bologna, IT)

**Introduction** The hypothesis of this study is that the use of a single tibial tunnel MAT arthroscopic technique without bone plugs for sub/totally meniscectomized symptomatic patients would reduce symptoms and improve knee function at minimum 3-year follow-up (FU).
**Materials and methods** Forty-six patients who underwent a meniscal transplantation (26 medial, 20 lateral; 33 male 13 female) were prospectively evaluated at minimum 36 ± 15.4 months after surgery. The average age at surgery was 37.0 ± 10.9 years (range, 15–55). The transplantation was performed using an arthroscopic bone plugs free technique with a single tibial tunnel plus “all-inside” meniscal sutures. The anterior meniscal horn was sutured to the capsule. FU included a VAS for pain, subjective and objective IKDC, Lysholm, Tegner and SF-36 scores. All patients underwent radiographic and MRI evaluation of the involved knee before the surgery, 30 of these (15 medial, 15 lateral) underwent a further MRI evaluation and at follow-up.
**Results** Regarding clinical evaluation, there was a significant improvement at FU: VAS ($p < 0.0001$), SF-36 ($p < 0.0001$ for physical component and $p = 0.0147$ for mental component), Tegner Activity ($p < 0.0009$), Lysholm ($p < 0.0001$), subjective IKDC ($p < 0.0001$) and objective IKDC ($p < 0.0001$). No significant difference was found between the medial and the lateral ones. There was no significant difference between patient with isolated and combined procedures. MRI findings showed 72% of extruded allografts. No significant difference in clinical outcome and modified Yulish score was found between patients with extruded allograft and with in situ allograft. MRI shows also a significant improvement of modified Yulish score ($p = 0.0002$ for femur and $p = 0.0215$ for tibia).
**Discussion** The results of this study are similar to the ones reported in the literature [1, 2], however these studies involve bone plugs and arthrotomic or mini-arthrotomic techniques.
**Conclusions** This study found that a single tibial tunnel MAT arthroscopic technique without bone plugs significantly reduced pain, improved knee function allowing patients to increase the activity level and protected cartilage at minimum 3-year FU.
**References**
1. Gonzalez-Lucena G, Gelber PE, Pelfort X, Tey M, Monllau JC (2010) Meniscal allograft transplantation without bone blocks: a 5- to 8-year follow-up of 33 patients. Arthroscopy 26(12):1633–1640
2. Verdonk PC, Verstraete KL, Almqvist KF, De Cuyper K et al. (2006) Meniscal allograft transplantation: long-term clinical results with radiological and magnetic resonance imaging correlations. Knee Surg Sports Traumatol Arthrosc 14(8):694–706

### Chronic lateral ankle instability treatment: retrospective study on 10 consecutive cases using modified Castaing surgical technique

M. Conte, S. Vassallo, A. Cadoni, S. Sechi, G. Piu*, F. Caputo, M. Salvi

Unità di Chirurgia Protesica e dello Sport, Casa di Cura Lay (Cagliari, IT)

**Introduction** Ankle sprains represent the 20% of all sports traumas. The 20–30% of acute capsulo-ligamentous lesions might turn into chronic instability. In Literature we can find over 70 surgical techniques and they are divided into two groups: anatomical and non-anatomical. With this retrospective study (Level IV) we have assessed clinical and X-ray exams results in 10 consecutive patients treated with the above surgical technique (graft with peroneus brevis semitendineous through peroneal tunnel).

**Materials and methods** Between February 2003 and September 2010, 10 patients were treated: mean age 34.6 years (min. 22–max. 45); 7 male (70%); right side, 90% of the cases. Agonistic sports activity 88.9%. Mean time between trauma and surgical operation 122.7 months (min. 15–max. 360). Mean f.u., 9 months (min 5–max. 94). The Clinical assessment was performed using AOFAS and KAIKKONEN score. AP and LL preoperative ankle X-ray e f.u. to assess an eventual arthritis evolution. Statistic assessment: use of Excel (Microsoft OFFICE 2010) and "Software designed for biomedical disciplines" 6/ed (2007).

**Results** the AOFAS started at 50 points (min. 24–max. 77) ending with 91.5 points (min. 84–max. 100); the KAIKKONEN started at 34 points (min.10–max. 70) ending with 88 (min. 60–max. 100; $p < 0.001$). The subjective functional result was excellent in 60% of the cases and good in 40% of the cases. The active and passive R.O.M. in flexion–extension was complete in 100% of the cases. A moderate limitation of the inversion was noted in 60% of the cases and it was severe in 20% of the cases, without relation with a degree of satisfaction expressed by the patient. The anterior drawer and the talar tilt tests proved negative in 100% of the cases. 8 patients practising agonistic sports activity: 6 same level, 2 lower level, 1 abandoned Sports' for other reasons. The X-ray assessment showed no signs of arthritis.

**Conclusions** The results obtained were encouraging and can be superimposed to those reported in Literature for the most sophisticated and most investigative anatomical techniques used. The simplicity of Castaing modified technique and the low incidence of complications drive us to see it as a first choice technique even on agonistic Sports athletes. The possible arthritis evolution linked to the excessive reduction of the astragalic tilt, which has not been considered in this study for its short time of follow-up, requires a re-assessment with a more appropriate follow-up.

### Does pre-operative laxity influence anterior cruciate ligament reconstruction outcome? A quantitative analysis by surgical navigation

T. Bonanzinga*, G.M. Marcheggiani Muccioli, N. Lopomo, C. Signorelli, S. Bignozzi, A. Grassi, A. Bondi, C. Musiani, M.P. Neri, M. Marcacci, S. Zaffagnini

Istituto Ortopedico Rizzoli (Bologna, IT)

**Introduction** A successful anterior cruciate ligament (ACL) reconstruction depends on the surgical procedure, rehabilitation process and patient's specific laxity. An isolated ACL lesion is a quite rare event, thus a correct intra-operative evaluation of combined lesions remains fundamental. This study aimed to retrospectively analyze knee laxity acquired by means of a navigation system in order to verify higher preoperative laxity values remained higher after isolated ACL reconstruction. Second, we analyzed the correlation between pre- and post-reconstruction values in order to identify laxity thresholds for pathological condition.

**Materials and methods** Knee laxities—namely anterior/posterior at 30° (AP30) and 90° (AP90), varus/valgus at 0° (VV0) and 30° (VV30), internal/external rotation at 30° (IE30) and 90° (IE90)—acquired on 115 patients before and after ACL reconstruction, were analyzed. Mimicking IKDC objective score, patients were classified in 4 groups according to the increasing level of laxity. Correlation analysis between pre- and post-reconstruction values and between pre-operative IKDC grading and quantitative pre-reconstruction classification was performed. Dichotomizing the groups, laxity thresholds for pathological condition were also defined.

**Results** Pre- and post-reconstruction classification revealed that the differences identified amongst groups before the reconstruction were maintained also after the surgery, even if the laxities were reduced. Pathological thresholds were identified as: 3.8° for VV0, 3.7° for VV30, 11.9° for IE30, 10.0° for IE90, 6.4 mm for AP30 and 6.0 mm for AP90. The correlations between pre- and postreconstruction laxity reported fair/good Pearson coefficients (r ranging between 0.50 and 0.82). The correlations between quantitative pre-reconstruction laxity classification and pre-operative IKDC objective grading revealed good Spearman coefficient only for Lachman test (rs = 0.71).

**Discussion** This study endorsed the hypothesis that those patients with higher pre-operative laxities, mainly due to combined lesions on secondary restraints, would maintain higher values also after isolated ACL reconstruction.

**Conclusions** The definition of laxity thresholds could be performed using navigation technology, thus guiding the surgeon to adapt the surgical strategy to patient-specific clinical requirements.

### Talar osteochondral lesion repair by arthroscopic autologous chondrocyte implantation: 5-year clinical and T2-maping MRI evaluation

M. Cavallo*, M. Battaglia, F. Vannini, R. Buda, A. Ruffilli, T. Buscio, L. Ramponi, S. Giannini

Istituto Ortopedico Rizzoli (Bologna, IT)

**Introduction** The ideal treatment of osteochondral lesion of the talus (OLT) is still controversial. Aim of this study was to evaluate clinically and by MRI T2-mapping the mid-term result (5-year follow-up) of a series of patients treated by autologous chondrocyte implantation (ACI) and investigate possible correlations with the clinical score.

**Materials and methods** Twenty patients, aged 31.4 ± 7.6 years and affected by OLT underwent arthroscopic ACI. The mean size of the lesions was 1.6 ± 0.9 $cm^2$. All patients were evaluated by clinical score (AOFAS score) and traditional MRI (Mocart score) pre-operatively and every 12 months up to a 63.2 ± 14.5 months follow-up. Finally the study was completed by MRI T2-mapping at final FU.

**Results** The pre-operatively AOFAS score was 57.2 ± 14.3 and it increased to 92.7 ± 11.4 ($p < 0.0005$). The best clinical outcomes were found in patients with more than 4 years follow-up. (Rho = 0.326, $p = 0.07$). By a radiographic point of view, two cases had arthritis progression at follow-up. MRI showed a good surface repair tissue and a completely filled lesion in 17 patients. In the regenerate tissue was highlighted the presence of hyaline cartilage with a mean T2 map value of 35–45 ms (10–98% of the repaired lesion area), cartilage in remodelling phase (1–60%) and fibrocartilagineous tissue (2–62%). Depth of the lesion negatively influenced the clinical score (R = −0.565, $p = 0.009$), the integrity of regenerate surface (Rho = −0.397 $p = 0.046$) and the percentage of filling of osteochondral defect (Rho = 0.424 $p = 0.034$).

**Discussion** MRI T2-mapping is an innovative tool to assess qualitatively the cartilage repair process, particularly interesting because it's a non-invasive technique able to provide information about the repaired area. These characteristics make T2 Map a valid alternative to bioptic harvest for evaluation of ACI in the ankle at 5-year follow-up.

**Conclusions** Clinical results demonstrate the validity of arthroscopic ACI in the ankle with a tendency of improvement of clinical score over the time, even if foci of fibrocartilage or tissue in remodelling phase were present in some cases. MRI T2-mapping integrated with Mocart score is a valid technique to evaluate regenerate tissue's morphology and quality. This tool also permitted correlations with clinical findings.

### Arthroscopic meniscal repairs: 135 patients analysis

E.C. Cenna*[1], F. Rosso[2], S. Spolaore[2], E. Graziano[1], L. Drocco[1], M. Crova[1]

[1]AO CTO-M. Adelaide, II Clinica Ortopedica (Turin, IT);
[2]Università degli Studi di Torino (Turin, IT)

**Introduction** The purpose of this study is to assess the medium-term survival of 140 meniscal sutures performed in 135 patients in the Orthopaedic Clinic II CTO Hospital of Turin.

**Materials and methods** 140 sutures (135 patients) were performed between June 2003 and July 2009. Two techniques were used: "All-inside" with Biostinger (ConMed®) in R–R zone of the posterior horn lesions and "Outside-in" with PDS wire for the injuries of the RR and RB areas and for bucket handle tears. The two types of suture were associated in the complex lesions. The post-operative protocol included the absence of loading for 4 weeks with flexion limited for 15 days at 60° and 90° for next 15 days and no squatting for 6 months. For the clinical evaluation Tegner and Lysholm score was used and in 16 cases an MRI was performed.

**Results** The average follow-up was 31.7 months and the average age was 28.5 years. In 133 cases the tears involved the medial meniscus. In 115 patients the meniscal repair was associated with an ACL reconstruction. In 14 cases on 16 MRI showed good repair of the lesion. We detected 17 cases of recurrence of symptoms of which 15 meniscal re-injury in the repaired area. In 11 of these patients the suture was associated with ACL reconstruction. At the second-look selective meniscectomy was performed. The remaining 6 patients did not require surgical treatment. Considering the failures according to the repair's technique, there were 8 cases of meniscal suture with PDS (of which 5 cases had only one point), 4 with Biostinger sutures, and finally, 3 mixed sutures. Splitting the failures based on the type of lesion, 9 out of 15 failures (60%) had lesions of the vertical 1/3 back to 1 to 3 cm and the remaining 6 cases (40%) of bucket handle tears. 33 patients were not satisfied and 25 of them did not return to previous levels of physical activity. The Tegner-Lysholm rating scale evaluation reported 91 excellent results, 21 good and 6 poor.

**Discussion** The failure rate of meniscal suture is 13%, relatively low compared to the literature. The tears' cuentation, applying multiple vertical sutures, spaced 5–6 mm between them, the age, the type and timing of the injury and the observance of a proper post-operative protocol, as well as the association with ACL reconstruction have fostered a good healing.

### ORAL COMMUNICATIONS

### *C37—BIOTECHNOLOGY IN THE FIELD OF TRAUMA 1*

### Reconstruction by MSCS of knee and ankle coin lesions: preliminary results

S. Zanasi*

Villa Erbosa Hospital, Gruppo San Donato e Domus Medica (Bologna, IT and San Marino, RSM)

**Introduction** Since 2006 Fortier et al. have shown that you can use a concentration technique of bone marrow cells, called BMAC in the operating room, and during the same session focused on implanting bone marrow with significant advantages in terms of cost and time, using the so-called "one-step surgery" cartilage for transplantation.

**Materials and methods** The technique consists of taking 60 ml of bone marrow aspiration from the ipsilateral iliac crest, using a disposable kit, and centrifuge it (Harvest PreP2 Smart System) 5–6 times to concentrate mesenchymal cells contained in the bone marrow. Through the use of thrombin from autologous blood collection, you can turn the bone marrow and produce a concentrated gel that is loaded in the suitably shaped scaffold (Hyaff-11) used to reconstruct the chondral lesion. Patients are then directed to a specific rehabilitation program: 55 patients (32 m, 23 f, mean age 37 ± 9) were treated from 2/2009 to 2/2011 for coin lesion with grade IV Outerbridge interesting patella, MFC, LFC, emi-tibial plate, talar dome sized from 3 to 9 $cm^2$ (4.6 $cm^2$ average).

**Results** The clinical evaluation at average follow-up of 14 months (range, 8–24) executed according to. ICRS-IKDC and AOFAS protocols showed, in comparison with preoperative data, a highly significant improvement ($p < 0.0001$). The evaluation of the cartilage by 1.5T NMR, performed at 1, 3, 6, 12 months, according to MOCART scoring system consistently documented the complete coverage of the loss of substance. The two 2nd looks at 12 months follow-up showed a complete reconstruction of the loss of substance with moderate hypertrophy of the regenerated tissue and histology showing high expression of type II collagen and proteoglycans.

**Conclusions** The clinical and NMR preliminary data documented that after inoculation of concentrated bone marrow (BMSCs) on a scaffold of esterified hyaluronic acid (Hyaff-11) the patient has cool, dry, painless knee, with complete ROM recovery; no local or systemic complication occurred; at 1, 3, 6, 12 months follow-up, 1.5T NMR imaging showed complete defect coverage by cartilage tissue like; thus resulting, in the short term follow-up, in ICRS and AOFAS scores comparable or improved versus the consolidated technique of ACT; furthermore the technique has the particular advantage of a single operative time and the significant cost containment.

### Achilles tendon tenorrhaphy and platelet rich plasma

P. Zedde*, A. Melis, A. Manconi, A.F. Manunta

AOU Sassari Clinica Ortopedica (Sassari, IT)

**Introduction** Actually Achilles tendon rupture is in increasing frequence in relation to the growing interest and participation of people of every age to sport activities. Tenorrhaphy represents the elective surgical procedure for this pathology; it can be realized with different surgical approaches, open or mini open (Achilon), or percutaneous (mini invasive- Ma-Griffth, Tenolig). The treatment of tendon rupture should be based on the knowledge and the comprehension of biological processes of tendon healing, which can be enhanced with Platelet Rich Plasma application.
**Materials and methods** From February 2008 to February 2010 we treated 21 patients with Achilles tendon rupture, repaired with termino-terminal suture by using a particularly strong suture named Fiber Wire. In 10 cases we added to surgical procedure PRP application in the side of injury; in 1 patient with bilateral simultaneous lesion, the left side was repaired just with the tenorrhaphy, while in the right side PRP gel was added, in order to eventually evidence any difference in lesion repairing process. Post-operative care was absolute rest for the first 3 weeks, with a semi-rigid bandage, weight bearing forbidden; followed by another 4–5 weeks in a rigid brace with partial weight bearing crutches assisted. From the 60° day complete weight bearing was allowed with gradually crutches non use for progressive functional recovery. Clinical data were assessed at 6 months and 1 year from surgery with the patient satisfaction and with a specific clinical score.
**Results** All patients regained normal range of motion, full functionality and return to normal daily and sportive activities. We achieved the complete recovery faster in patients treated with platelet gel, as we can appreciated in the case of bilateral rupture, where the tendon treated with PRP showed a faster healing.
**Conclusions** A moderate mechanical stress promotes the progressive disposition of collagen fibers along the direction of muscle force lines, optimizing the tensile resistance. The mobilization and the early weight bearing have a positive effect on tendon reparative process. Actually the use PRP allows the local increase of growth factors's concentration, optimizing the healing process and a faster functional recovery without recurrence of lesion.

### Enhanced recovery of disturbs in bone healing of lower limb shaft fractures by using PRP

C. Angrisani*, S. Del Prete, G. Roccasalva, L. Bifulco, E. Taglialatela, P. Greco

AORN Azienda Ospedaliera S. Sebastiano (Caserta, IT)

**Introduction** After a revision of femoral and tibial fractures from 1997 to 2010, we evaluated complications as pseudoarhtrosis and bone loss substance in our surgical treatment.
**Materials and methods** The revision concerned closed and exposed fractures which showed a difficult healing of bone. We used external fixation and intramedullary nail, sometimes plates and screws. 1081 patients with a multiple trauma and exposed fractures, often comminuted, were operated. 20% of cases needed another operation, in 16% of cases cortical and spongious bone graft as PRP was applied; the latter resembles a gel added to fracture bone during surgical act or applied through skin by 18G needle. In 123 cases we had delayed fractures, 173 cases infected pseudoartrosis recovered tanks to $O^2$ therapy in iperbaric room and surgical toilette and with substitution of sintesi; in 53 cases non-infected pseudoartrosis occurred and were treated by opening and cruentation of bone using intramedullary nail. Follow-up was on average 36 months and patient examination satisfied bioclinical and radiologic criteria.
**Results** We reviewed 137 cases of delayed and pseudoartrosis fractures. We observed a complete resolution in 70% of cases with good results of healing bone, in 30% of cases wev had bad results that ebnded in osteomielitis or bone amputation or lesser lengthening of 7 cm.
**Discussion** In order to reach satisfying results, correct timing and use appropriate and modern devices is mandatory. Despite surgical solutions, prescribed according protocols proposed by many renowned authors, these devices may produce complications that do not allow easy recovery and lead to pseudoartrosis. In our work we underline the important use of modern techniques as PRP which is prepared by autologous blood of the patient and added to healing fractures favouring easier recovery from delayed unions and pseudoarthrosis by stimulating the osteoblasts to bone production.
**Conclusions** In conclusion we really need to do a correct reduction and stabilization of fractures and associate use of PRP to prevent major complications and enhance fracture healing and quick recovery, allowing an acceleration of functional rehabilitation after the surgery.

### Analysis system biotechnology cervico-cephalic nailing with single and double screw used in the treatment of lateral fractures of the femoral neck

V. Caiaffa*, A. Fraccascia, V. Freda, R. Cagnazzo

S.C. Ortopedia e Traumatologia, Ospedale SS. Annunziata (Taranto, IT)

**Introduction** The gold standard in the treatment of pertrochanteric fractures, evidenced by bibliographical results, appears to be the intramedullary nailing, because it allows a good functional and quickly recovery of the patient, preventing the complications of the setting. However, there arises the question of what is the best system: the single or double cephalic screw.
**Materials and methods** There are two computer models to study the difference between a single screw system and a double one, attempting to maintain the same size (although the system with a single screw has a a larger diameter).
**Results** The double screw system showed a more functional distribution of the load because the neutral axis is equidistant and external to the section of screws. This allows using all the available material. On the other hand, in the single screw system the neutral axis passes through the screw itself, by making use of large diameter.
**Discussion** The bending load distribution between the two screws is optimized and the rotation of the femoral head is neutralized.
**Conclusions** We report the results of a study of performance of cervico-cephalic nails with single and double screw. In this laboratory work is complemented clinical outcomes.

### High energy shock waves for the treatment of delayed unions

S. Scarponi*

Istituto San Donato (Milan, IT)

**Introduction** Focused shock waves have been reported to induce trabecular and cortical bone formation. Possible mechanisms are: increased growth factors, stimulate mesenchymal stem cells, triggered bay damage periostal or cortical and trabecular bone.

**Case report** A 32-year-old man treated for distal tibia fracture 43-A3, AO classification, by LCP plate system. Eight months after, X-rays showed delayed union. At a second intervention, bone graft from iliac crest was used.
**Results** Ten months after, X-rays showed union of tibial fracture. Treatment focused on extracorporeal shock waves (16 MHz) for 5 weeks, 2 times a week. Three months after, the fracture was healed with new bone formation.
**Discussion** The distal tibia fractures represent 10% of all tibia fractures and they are the result of high energy traumas, characterized by bad prognosis, as in this case.
**Conclusions** A double or prolonged treatment (5 weeks) with focused extracorporeal shock waves increase bone tournover in side of fractures, improve cancellous and cortical bone architecture and mechanical properties.

### *C38—BIOTECHNOLOGY IN THE FIELD OF TRAUMA 2*

### Humerus PSA with critical bone loss: the role of biotechnology

G.M. Calori*, M. Bucci, P. Fadigati, E. Mazza, M. Colombo, C. Ripamonti

C.O.R., Istituto Ortopedico G. Pini (Milan, IT)

**Introduction** NUs and critics bone defects of the humerus are a very disabling complication in upper extremity trauma. The localization of these diseases (proximal epiphyseal, diaphyseal or distal epiphysis) and the general conditions of the patient, soft tissue and bone determine the choice of treatment and the possible application of biotechnology. Where possible, in proximal epiphyseal lesion, a plate and screw reconstruction with the implant of biotechnology in polytherapy is arguable. In diaphyseal lesion, it is necessary to quantify bone defect, and if it is less than 4 cm, it is possible to perform oblique shortening osteotomy segment and applying a compression plate in a fixed-angle. Where living conditions are poor it is always possible to apply the polytherapy and possibly increasing the stability of the system with the implant of allograft from the bone bank. In distal epiphyseal lesion, instead, it is often necessary to reconstruct the joint surface, even in these cases, the implant of biotechnology in polytherapy seems to be a good surgical choice. The purpose of this study is to analyze the cases of NU and of critical bone defects of the humerus treated in our department either with or without biotechnology dividing them according to the topography of lesion.
**Materials and methods** We analyzed 28 PSA and bone defects of the humerus, of these 14 patients (3 proximal epiphyseal, 8 diaphyseal, 3 distal epiphyseal) were treated without Biotechnology (*Group A*) and 14 (4 proximal epiphyseal, 9 diaphyseal, 2 distal epiphyseal) biotech-treated (*group B*). The results were analyzed by a clinical and radiographic follow-up period of 24 months.
**Results** The cure rates were 71.4% in *group A* both in terms of clinical and radiographic evidences against 85.7% in *group B*. All failures (4/14) in *group A* had the disease in diaphyseal epiphysis. The failures of the *group B* (2/14) instead were located distally at the first and second level in the epiphysis.
**Conclusions** Based on our results we can conclude in favor of surgical treatment of oblique shortening osteotomy reducing the shortening of the segment and the association of biotechnology in polytherapy (rh-BMP-7 + MSC + allograft) in the treatment of PSA and critical diaphyseal bone defects of the humerus. When both proximal and distal epiphyseal are affected, given the paucity of cases handled, it is not possible to conclude whether the application of biotechnology can be recommended, further investigations are needed to validate its effectiveness.

### Early results in osteosynthesis of fractures of the proximal humerus with a radiolucent plate of Peek

P. Budassi*, S. Stroppa

Azienda Ospedaliera (Cremona, IT)

**Introduction** The treatment of the fractures of the proximal humerus is controversial and there is a poor consensus especially for the three- and four-part fractures. The poor consensus concerns also the materials of fixation. We report the short-term follow-up of this kind of fractures treated by angular stable plate of Peek (Polyether ether ketone) fixed by titanium screws (Ti6AI4 V).
**Materials and methods** This non-randomized prospective study includes 10 patients with fractures of the proximal humerus and was started in December 2009. The average age was 70 years (range, 64–78). Of these, seven had 3-part fractures and three had 4-part fractures. Surgery was performed in the beach chair position using a delto-pectoral approach without detachment of the deltoid and an angular stable plate of Peek (DiPhos H—Hit MedicaR). Patient re-evaluation was performed following the Constant score.
**Results** The mean follow-up was of 10 months (range, 7–16). The mean Constant Score was 68 and the maximum was 84. The average front elevation was 98 degrees and the average external rotation was 28 degrees. There were no infections, malunions or loss of reduction in any of the patients. Union occurred in all patients at an average of seven to nine weeks postoperatively.
**Discussion** Early results in osteosynthesis of fractures of the proximal humerus with plates of Peek are encouraging. The ease of implantation and the percentage of union have been similar to those obtained with Titanium Plates. Also the functional outcomes have been similar to those obtained with ostheosynthesis using the traditional angular stable plate of Titanium. We also had the advantages provided by the new material. The hole in the fixed-angle plate in fact is not threaded, but the titanium screw that tap the plate hole, thus it can be inserted in the desired direction. Furthermore, the Peek is an inert material and so the galvanic corrosion between the plate and the screw head is absent: the removal of the implant is easier. Finally, the radiolucency of the plate is useful in controlling intraoperative reduction of the fracture and at follow-up of the healing process.
**Conclusions** The Peek seems to be a very interesting material in osteosynthesis. However, due to the short follow-up and the small number of patients, the proceedings and the results must be considered provisional.

### Diaphyseal forearm fractures: today treatment

N. Galvano*, M. Ferruzza, G. Caradonna, A. D'Arienzo

Clinica Ortopedica e Traumatologica, Policlinico Universitario (Palermo, IT)

**Introduction** Diaphyseal forearm fractures are approximately 10% of all fractures. In 15% of cases, they can be associated to a "Monteggia" or "Galeazzi" fracture-dislocation, "Essex Lo Presti" type lesion is instead quite rare. According to our experience, the best surgical treatment is the plate and screws fixation, reserving the external fixator to cases with compromised skin.

**Materials and methods** From January 2006 to December 2010, we observed 32 diaphyseal forearm fractures. In 10% of cases, they were associated with a Monteggia type dislocation, only the 3% with a Galeazzi lesion. Direct or indirect traumas are the damaging mechanisms, usually as the result of road accidents. All cases were treated with fixed-angle plates and screws. Fractures were classified according to the complexity through AO classification and to the stadium of exposure through the Gustilo Andersen classification. After the surgical treatment, antibiotic and antithrombotic therapies were performed with immobilization in a plaster cast.
**Results** The used technique allowed us to achieve positive results in 90% of cases, about fracture healing and functional recovery. A radiological assessment was performed at 3, 6, 12 months after surgery. We used the DASH scale and VAS visual scale to valuate the clinical and functional recovery. All patients underwent magnetic therapy a month after surgery and this treatment accelerated the bone healing.
**Discussion** Diaphyseal forearm fractures represent a difficult problem for the surgeon due to the most frequent complication: the pseudarthrosis. Treatment with fixed-angle plates plus the magnetic therapy leads to bone healing in 90% of cases, with any associated dislocation.
**Conclusions** According to our experience, the described treatment shows a complete bone healing in 12 months after fracture.

### Kyphoplasty in spinal fractures with resin-like material "PMMA" and biological material "CERAMENT": differences, comparison, results and follow-up

M. Dragani*[1], S. Marcia[2]

[1]ASL di Pescara (Pescara, IT);
[2]Università di Cagliari (Cagliari, IT)

**Introduction** There are two classes of materials used in kyphoplasty resins or PMMA and biological materials. There are differences either in their use and indications or in the results. The resin-like material is permanent, the biological one is absorbable and it forms bone. CERAMENT is composed of hydroxyapatite and calcium sulphate, it does not increase in temperature, it is painless and it can be used in patients below the age of thirty-six. Once injected it stimulates osteoblasts attraction and bone formation. The aim of this study is to present indications, methods of use and preliminary results at one-year and its different use in Kyphoplasty.
**Materials and methods** Kyphoplasty was performed with a standard tool-Medtronic, and Kit One Step Express CDS. The patients were operated under local anaesthesia through unilateral access with scopica guidance. The PMMA material and barium sulphate injected are permanent. The CERAMENT is made with hydroxyapatite-calcium phosphate and iodine so that it is visible. Subacute and chronic vertebral fractures were treated and they were treatment/bust resistant. Controls were performed using VAS and ODI scale, radiography and CT HR. We made a comparison among 100 patients, 50 treated with PMMA and 50 patients treated with CERAMENT.
**Results** There were satisfactory results in VAS and ODI values. The scores were lower after the procedures with substantial differences between the groups. As far as CERAMENT is concerned the X-rays documented a reduction in visibility, the TCHR showed density values higher than adjacent bone. Controls of patients treated with PMMA documented the persistence of the resin. There were no major complications.
**Discussion** Kyphoplasty offers advantages over vertebroplasty. The advantages of CERAMENT are evident in any range of age, absence of pain during injection, reabsorption, bone regeneration, pain elimination/relief and stabilization of the collapse.
**Conclusions** The availability of different materials in the treatment of osteoporotic and traumatic vertebral fractures allows fulfilling several needs.

### Optimization of bone repairing biological response in forearm fractures treated by O'Nil internal fixation system

G. Pristerà*, N. Ruggieri, C. Palumbo

SCDO Ortopedia e Traumatologia, AOU San Giovanni Battista Molinette (Turin, IT)

**Introduction** The past experience with the use of plate osteosynthesis traditionally emphasized long consolidation times and many tears of fixation devices. In order to solve these problems, the authors introduce the use of the O'Nil internal fixation. This system is based on biological conditions of compliance with the periosteum and the cortical blood flow. This can be achieved only through technological devices: (a) the positioning of the plate differing from the bone surface; (b) the configuration of the tapered shape of mating screw-plate, reaching an angular stability system, extremely effective in transmitting the load forces, without screws mobilization and plaque rupture. Moreover, these features make easy a removal of the fixation itself.
**Materials and methods** From 2007 to 2009 the following cases were treated by O'Nil internal fixation system: 19 closed fractures of the forearm (4 women, 15 men, aged between 16 and 70 years); 11 polytraumas; 3 polyfractured cases; 5 cases with only forearm fracture (AO classification was: 5 22A1—22A2 2—3 22A3—2 22B1—22B2 2—2 22 B3—1 22C1—22C3 2); 5 fractures involving both bones; 6 ulna fractures, 8 radius fractures. 24 O'Nil forearm devices (3.5 mm thickness) were used.
**Results** We detected good results by: (a) X-rays signs of bone callus within a mean time of 55 days; (b) an early post-operative articular active recovery; (c) no devices intolerance. The only plaque rupture is due to a technical error in implanting. The only difficult removal was due to the excessive rigidity of first production plates in the mating screw/plate, which was promptly corrected in the last generation plates.
**Discussion** The cornerstones of this system are: (a) the greatest care to the periosteum, which should not be detached for the bone even in spite of a not perfect reduction; (b) the stability achieved between the plate and screws, without compressing the plaque on the bone surface, as it occurs in the traditional synthesis. These two conditions facilitate the natural bone repairing processes.
**Conclusions** The O'Nil internal fixation system has proven to be: (1) effective in optimizing the timing of bone repairing and early functional recovery; (2) easy and versatile system equipped with full instrumentation (3) stable not requiring any bone graft support; (4) non-invasive and well tolerated procedure for the patient.

### Using biotechnology in the outcome of traumatic calcaneal fractures

G.M. Calori*, M. Bucci, P. Fadigati, E. Mazza, M. Colombo, C. Ripamonti

C.O.R., Istituto Ortopedico G. Pini (Milan, IT)

**Introduction** Calcaneal traumatic fractures are difficult to be treated due to their complex segmental anatomy and to the special and delicate bone structure that characterizes this district. Patients who come to the attention of the expert in the field, often have had multiple surgeries with remaining inconclusive results and very debilitating complications of traumatic expression. Vicious consolidation or nonunion are are possible complications often characterized by severe bone loss that is replaced by abnormal tissue, no longer active and often necrotic, that must be removed. Defects secondary to axial vicious segmental consolidations that change the biomechanics of the foot and ankle joint, and the support and the kinetics of the step are also recurrent.

**Materials and methods** In our retrospective study we have critically analyzed 5 cases of traumatic outcomes of calcaneal fracture whose reconstruction was achieved by grafting xenograft: a valid therapeutic choice although not free of complications. The surgical protocol followed in this case is the following: corrective osteotomy with the possible removal of diseased tissue, plant material osteoconductive (scaffolds enriched autologous mesenchymal cells) and osteoinductive (growth factors), mechanical stabilization achieved by osteosynthesis with plate and/or staples.

**Results** In our, even though limited, experience we obtained encouraging results on the use of such biotechnology. We achieved in all cases an excellent osteointegration of the implant with complete healing processes, a good correction of the anatomical shape and calcaneal axis, a plantigrade support and a good joint functionality without residual functional pain. In one case we had to restore the application of advanced dressings and Vac-Therapy to overcome the suffering skin caused to fixation of the material used.

**Discussion** The plant of biotechnology is made of polytherapy on the principle of "Diamond Concept" which has four distinct elements: a stable biomechanical environment, cells, scaffolds and growth factors. This concept was further supplemented with a fifth element: the vascularization. The treatment aims to create a "biological chamber", a microenvironment in which all the necessary elements to bone regeneration can be found.

**Conclusions** Biotechnology is a powerful tool, validated by numerous national and international studies that emphasize the effectiveness and safety. Strict indication for surgery and a complete radiological study and a proper pre-operative planning are a priority in order to achieve a proper usage and avoid wastage or ill use.

### *C39—BIOTECHNOLOGY IN THE FIELD OF TRAUMA 3*

### Low intensity pulsed ultrasounds for bone healing: results of an observational, prospective, multicenter, Italian study

C.L. Romanò*[1], E. Meani[2], M. Caporale[3], G. Falzarano[4], M. Imbimbo[4], A. Toro[5], A. Kirienko[6]

[1]Istituto IRCCS Galeazzi (Milan, IT);
[2]Istituto Gaetano Pini (Milan, IT);
[3]Ospedale Civile Monterotondo (Rome, IT);
[4]Ospedale Civile di Benevento (Benevento, IT);
[5]Ospedale Civile Mercato (San Severino, IT);
[6]Istituto Clinico Humanitas (Rozzano-Milan, IT)

**Introduction** The M.M.O.S.S. (MicroMechanical Osteogenetic Stimulation Study) is an observational, prospective, multicentre study, performed in Italy from 2005 to 2009. Seven centers participated in data collection, in order to assess the rate and timing of clinical recovery and bone healing in patients with fresh fractures of bone nonunions, treated with low-intensity pulsed ultrasound (LIPUS).

**Materials and methods** In all cases, treatment with LIPUS was performed with the FAST unit (IGEA SpA), which provides a pulse frequency of 1.5 MHz, with average power at the site of treatment of 30 mW/cm$^2$. Criteria for inclusion in the study were: patients with recent fractures or bone non-unions, requiring treatment with LIPUS, in the opinion of the physician. Exclusion criteria were: unstable or displaced fractures, tumors, pacemaker, patients not able to continue treatment at home. For all patients were collected clinical data and radiographs before surgery and at follow-up, indicating the demographic data, characteristic of the fracture, type of treatment, risk factors, bone healing. 144 patients were included in the study, 12 were lost to follow-up. Of available patients to final follow-up 86 were nonunions and 46 fresh fractures.

**Results** The mean age of the group of patients with non-union was 46 ± 19 years; 87% of non-unions reached radiographic healing, with an average treatment time of 108 ± 71 days. Overall 34% of the patients were smokers, whereas in the group of not-responders to treatment the percentage of smokers rose to 80%. In the group of fresh fractures, the average age was 45 ± 24 years. The cure rate was 95%, with an average treatment time of 52 ± 28 days.

**Discussion** This multicenter, prospective observational study show that LIPUS allows to achieve bone healing in a heterogeneous group of non-unions and fresh fractures, respectively in 87% and 95% of cases.

**Conclusions** Treatment with pulsed ultrasound is simple to perform and totally innocuous and can be run directly by the patient at home, even in the presence of fixation or bone infection. Risk factors specific to the patient, such as cigarette smoking, seem to be strongly correlated with the prognosis.

### Delayed unions/nonunions: treatment with shock waves

A. Carfagni, G. D'Innella*, F. Clerici Bagozzi

Rome, IT

**Introduction** The aim of this study is to examine the effects of shock waves (ESWT) in the defects of bone healing.

**Materials and methods** In the period between March 2002 and January 2011 at the Hospital San Carlo-IDI in Rome 24 cases of delayed unions and 69 cases of nonunion were treated with shock wave therapy. The sites covered were: 20 tibias, 13 humerus, 3 clavicle, 6 tibias and fibulas, 11 femurs, 11 ulnas, 2 radius, 12 carpal scophoids, 4 radius and ulnas, 3 metacarpals, 4 olecranons, 3 fifth metatarsals,1 tarsal cuboid. The average age was 51 years (range, 18–84 years). The used equipment was an electromagnetic lithotripter with ultrasound probe and coaxial arc C radiological MODULITH SL-K and DUOLITH of STORZ MEDICAL. The treatment protocol included a series of 4/5 sessions at a rate of twice per week, each session was provided with 3200 shots at a frequency of 240 strokes per minute with an energy level between 0.20 and 0.55 mj/mm, and we prescribed to the patient no bearing, when necessary, rest and a cast to immobilize the segment. The follow-up: X-rays at 30–40 days after the last session.

**Results** We treated 93 patients with thess results: complete healing 55% (51 pts), partial healing 27% (25 pts), nonunions 18% (17 pts).

**Discussion** Extracorporeal shock-wave therapy is not effective as surgery in stimulating union of long-bone hypertrophic nonunions, but it could be used to stimulate the healing process in delayed unions/nonunions.

**Conclusions** The high percentage of success suggests this method, non-invasive and repeatable over time, as the treatment of choice in delayed union and nonunion in combination with surgery or not.

### Pridie's marrow stimulation technique combined with collagen matrix and marrow implementation for cartilage repair: a study in still growing sheep model

M. Giordano*[1], A.G. Aulisa[1], A. Gigante[2], P. Savignoni[1], G. Isacchi[1], F. Zinno[1], V. Guzzanti[1]

[1]Ospedale Pediatrico Bambino Gesù (Rome, IT);
[2]Clinica Ortopedica, Università di Ancona (Ancona, IT)

**Introduction** Articular cartilage lesions are common both in adults than in growing age. Untreated large critical full-thickness defects (FTD) progress to degenerative alterations and than to arthritis. The management of these lesions continues to be problematic for orthopaedic surgeons. This study assessed the repair of FTD in not mature and still growing sheep model and compared repaired tissue after Pridie's technique combined with type I collagen matrix to the tissue obtained using the same technique combined with autologous bone marrow concentrate.

**Materials and methods** For this study we used ten still growing sheep. Using a hand drill, 2 large critical size FTD 6 mm in diameter was created in the medial femoral condyle of the left knee of each animal. A lesion was treated with perforations in the subchondral bone according to Pridie's technique combined with type I collagen of the membrane, sealed in the peripheral border with BioGlue (*group 1*). The second lesion was treated using the same technique but with the addition of autologous bone marrow concentrate taken from iliac crest (*group 2*). Each specimen (follow-up 12 months) was subjected to macroscopic, histological and immunohistochemical examination.

**Results** Results refer to macroscopic aspects of surface of lesion and histological features. Macroscopic evaluation documented a complete repair of the lesion in all specimens. Both lesions in *group 1* and those in *group 2* showed a good continuity between the reparative tissue and the adjacent healthy cartilage. In both groups the histological appearance of the reparative tissue was similar to the normal surrounding hyaline cartilage with values ranging from 11.7 to 15.2 according to ICRS visual assessment score scale.

**Discussion** The bone marrow stimulation techniques are based on the biological principle of recruitment of mesenchymal cells from marrow cavity. The matrix scaffolding prevents the dispersion of osteochondral progenitor cells from bone marrow cavity and facilitates the invasion, proliferation and organization of these, while maintaining the exchange of solutes with the articular cavity.

**Conclusions** Pridie's technique combined with type I collagen matrix achieves complete repair of a large critical FTD in a not mature and still growing sheep model. The histological features of the reparative tissue are similar to hyaline cartilage. The addition of autologous bone marrow concentrate does not improve markedly the quality of histological features.

### Demineralized bone matrix (DBX) in nounions and bone defects treatment

A. Rossi, A. Rocca, F. Cividini*

Cliniche Humanitas Gavazzeni (Bergamo, IT)

**Introduction** Despite modern osteosynthesis systems reduced nonunion incidence from 10 to 4–8% nowadays, its cure remains difficult because of technical and biological problems and because of length and costs of treatments. How can we reactive an osteogenetic bone process which seems to be exhausted?

**Materials and methods** We treated 20 patients with demineralized bone matrix, with diagnosis of nonunion in 17 cases, one single case of aneurysmatic bone cyst, one of bone healing delay and one case of femural head necrosis. In 16 cases we needed to replace ostheosyntehesis devices for failure or breakage. In 4 cases we used a single demineralized bone matrix graft as a starter for bone healing in a favourable mechanical environment or as an osteoinductive filler (aneurismatic bone cyst).

**Results** DBX is a mix of demineralized bone matrix (DBM) from human donor and a hyaluronan matrix as a biocompatible carrier, completely rehabited by host bone in 4–6 months. It appears to be composed of collagene and growth factors (BMPs) which are responsable of the osteoinductive skills. BMP-2 alone promotes osteoblastic differentiation of mesenchimal cells without any multiplication. The presence of DBM allows multiplication of mesenchimal cells and their differentiation on osteoblastic way.

**Discussion** Where the osteosynthesis devices appeared to be stable, the use of DBX only leaded to bone union and clinical healing (3 cases). Clinical healing was achieved in two young patients through mixing DBX and mesenchimal stem cells. The other cases presented a bad mechanical environment with mobilization or breakage of devices with axial defects: in these case we performed a new and stable synthesis. Grow factors and bone substitutes grafting combined to a stable synthesis in respect of Giannoudis' diamond criteria leaded to an osteogenetic process and bone healing.

**Conclusions** In our clinical experience DBX has been showed as a valid help in treatment of pathology which require a regeneration pulse for bone healing.

### Polytherapy in "borderline" cases of PSA and loss of substance

G.M. Calori*, M. Bucci, P. Fadigati, E. Mazza, M. Colombo, C. Ripamonti

C.O.R., Istituto Ortopedico G. Pini (Milan, IT)

The treatment of nonunion (PSA) of long bones and bone defects remains a critical challenge for trauma surgeon often with unsatisfactory results and long morbidity. There are "borderline" cases difficult to be treated, requiring a careful use of resources, a strict indication and proper surgical technique for their complexity. They're characterized by: significant loss of substance, multiple previous surgeries, presence of atrophic or necrotic bone, broken fixation devices, attempts to fill (previous grafts and cement), severe deformities, poor soft tissue conditions and general condition of the patient (diabetes, smoking, infections) that slow or prevent the physiological healing. In all these cases the process that is undertaken is often characterized by a number of specialist visits, radiological studies, new surgical inconclusive attempt with a high social and health costs. In addition drastic decisions such as arthroplasty, arthrodesis or amputation may be indicated valid forms of transport with external fixation or the new techniques of surgical reconstruction with bank bone grafts and synthesis associated with biotechnology. Today we have good support (growth factors, scaffolds, mesenchymal stromal cellular) that can be used to replace autologous graft. They are effective, but expensive, therefore, require a strict indication and proper use of the surgical site (right application and good and stable osteosynthesis) in order to avoid unnecessary or ill-uses. Especially in those "borderline" cases in which local and general conditions are sometimes so severe that it may discourage use.

### Genetic effects of pulsed electromagnetic fields (PEMFS) on human osteoblast-like cells (MG-63) in vitro

V. Sollazzo*[1], F. Carinci[2], A. Palmieri[3], F. Pezzetti[4], L. Massari[1]

[1]Clinica Ortopedica, Università di Ferrara (Ferrara, IT);
[2]Istituto di Chirurgia Maxillofacciale (Ferrara, IT);
[3]Laboratorio di Chirugia Maxillofacciale ed Ortopedia, Università di Ferrara (Ferrara, IT);
[4]Istituto di Istologia ed Embriologia Generale (Bologna, IT)

**Introduction** Although pulsed electromagnetic fields (PEMFs) are used to treat delayed unions and nonunions, their mechanisms of action are not completely clear. However, PEMFs are known to affect the expression of certain genes. We asked whether PEMF affect gene expression in human osteoblastlike cells (MG63) in vitro, and whether and to what extent stimulation by PEMFs induces cell proliferation and differentiation in MG-63 cultures.
**Materials and methods** We cultured two groups of MG63 cells. One group was treated with PEMFs for 18 h whereas the second was maintained in the same culture condition without PEMFs (control). Gene expression was evaluated throughout cDNA microarray analysis containing 19,000 genes spanning a substantial fraction of the human genome.
**Results** PEMFs induced the upregulation of important genes related to bone formation (HOXA10, AKT1), genes at the transductional level (CALM1, P2RX7), genes for cytoskeletal components (FN1, VCL), and collagenous (COL1A2) and noncollagenous (SPARC) matrix components. However, PEMF induced downregulation of genes related to the degradation of extracellular matrix (MMP-11, DUSP4).
**Discussion** In this study we report for the first time in the existing literature the genetic portrait determined by PEMFs exposure in human osteoblast-like cells. PEMFs appear to induce cell proliferation and differentiation. Furthermore, PEMFs promote extracellular matrix production and mineralization while decreasing matrix degradation and absorption.
**Conclusions** Our data suggest specific mechanisms of the observed clinical effect of PEMFs, and thus specific approaches for use in regenerative medicine.

### Use of platelet-leukocyte membrane in arthroscopic repair of large rotator cuff tear: a prospective randomized study

S. Gumina*[1], S. Carbone[1], P. Albino[1], V. Campagna[2], F. Postacchini[1]

[1]Università di Roma “Sapienza” (Rome, IT);
[2]Ortopedia e Traumatologia, Ospedale Militare Celio (Rome, IT)

**Introduction** Arthroscopic cuff repair generally provides satisfactory results, in terms of shoulder pain decrease and improvement of range of motion. Unfortunately, imaging studies demonstrate that with the available arthroscopic techniques re-rupture rate is high. The use of autologously prepared platelet-leukocyte membrane is a new method which might stimulate and accelerate soft tissue healing. The purpose of this study was to evaluate the clinical and MRI results of arthroscopic rotator cuff repair with and without the employment of platelet-leukocyte membrane in patients with large postero-superior cuff tear.
**Materials and methods** We enrolled 80 consecutive patients with a full-thickness large supero-posterior cuff tear. All the lesions were managed with an arthroscopic single row repair. Patients were randomized and treated either with or without platelet-leukocyte membrane inserted between the foot-print and the tendon. When membrane was used, we utilized one membrane for each anchor. All patients were pre and postoperatively assessed using the Constant score and SST. MRI was performed before the operation and at the follow-up. Images were acquired for structural and qualitative assessment of the rotator cuff and repair integrity was determined according to Sugaya’s classification. A statistical analysis was performed.
**Results** Comparison between groups did not show significant differences for each independent variable except for age and pre and post-operative shoulder pain level that resulted statistically different between the two Groups. Activities of daily living, range of movement and power do not have an impact on the difference between the two Groups. At follow-up, we observed cases of cuff re-ruptures only in the group of patients where the membrane was not used. Analogously, in this group an insufficient thickness without discontinuity of the repaired tendon was more frequently observed. The use of the membrane provided better results in terms of repair integrity ($p = 0.04$).
**Discussion** In our study, the use of the platelet-leukocyte membrane improved repair integrity compared with single row technique without membrane. This improvement does not seem to determine a better functional effect. In fact, the Constant scores, calculated in the two Groups, were similar when deprived of the shoulder pain component.
**Conclusions** The use of the platelet-leukocyte membrane may improve rotator cuff repair integrity.

### *C40—BIOTECHNOLOGY IN THE FIELD OF TRAUMA 4*

### Iliac crest harvested bone marrow centrifugate, allograft and platelet derived gel in trauma: our experience with a technique of intraoperative tissue engineering

V. Denaro*[1], A. Di Martino[1], G. Vadalà[1], M.C. Tirindelli[2], R. Papalia[1], C. Nobile[2], G. Rizzello[1], G. Avvisati[2]

[1]Cattedra di Ortopedia, Università Campus Bio-Medico di Roma (Rome, IT);
[2]Cattedra di Ematologia, Università Campus Bio-Medico di Roma (Rome, IT)

**Introduction** Orthopaedic applications in tissue engineering procedures commonly involve the use of scaffolds, cells and growth factors. We intraoperatively used allograft (from bone or tendons) as a scaffold, associated with bone marrow mononucleated cell concentrate harvested from the iliac crest and implemented with growth factors derived from PRP. Referring to our experience in the field of spinal fusion, we report the results of our experience with the use of this rescue technique in trauma surgery in patients with multiple comorbidities as well as with those with previous failed surgeries.
**Materials and methods** A volume of 60 mL of marrow blood was harvested from each patient and processed in a double chamber centrifuge container (Smart-Prep, Harvest Technology®). Red blood cells and platelet-poor plasma were separated at the end of the centrifugation from the cellular concentrate (10 mL). The fresh frozen corticocancellous bone or tendon allograft were purposely prepared and used as scaffolds for the reconstructive procedures. The platelet-enriched autologous fibrine glue (Vivostat®) acted as an adhesive and as a carrier of growth factors.
**Results** We observed in the bony targeted procedures the healing of the fusion or the healing of previous non-union, despite the presence of multiple comorbidities or the failure of previous treatments. For tendon targeted procedures, patients gained recovery of the segmental joint function.
**Discussion** Tissue engineering techniques require the integration of osteoconduction, osteoinduction and osteogenesis. Mesenchymal Stem Cells (MSC) are a rare bone marrow population with the property of self-renewing and differentiation into different cellular lines. This cellular population can play a fundamental role in tissue healing, and in the orthopaedic field it represents a potential cellular substrate for bone regeneration. The paucity of these cells leads to the use of manipulation procedures in order to concentrate them. The contemporary action of the growth factors from PRP can contribute to the success of the surgery. Using the allograft as scaffold takes the advantage to give to the processed cells a structural environment similar to the native tissue.
**Conclusions** In our casuistry, this intraoperative tissue engineering procedure, consisting of the use of allograft as scaffolds associated with bone marrow concentrate of mononucleated cells harvested from the iliac crest, plus growth factors derived from PRP, showed good versatility in the rescue procedures of patients with previous procedure failures as well as with those with multiple comorbidities.

### Antibacterial activity of platelet gel: in vitro study and comparison with literature

J.M. Taglioretti*, B. Osnaghi, F. Ierna

Ospedale Fornaroli (Magenta, IT)

**Introduction** Sepsis prevention in orthopaedics is a challenge which has got hardier and hardier. Since Osler in remote 1886 contradictory scientific evidences on the platelet gel (PLG) role against infections appeared. Platelet aggregation could favour bacteria adhesion, protect pathogenous from antibiotic activity and neutrophile clearance, while lack or disease of platelet would correlate with the risk of higher incidence and gravity of infections. (M.R. Yeaman 2010). The recent discovery of new activating platelet receptors the antibacterial answer and the degranulation of a series of microbicidal peptides (PMP) makes platelets immune effective and modulating.
**Materials and methods** At Magenta Hospital an in vitro study aiming at estimating the antibacterial efficiency of PLG towards bacterial stocks was carried out by a co-operation among the Department of Orthopaedics, the Transfusion Service and the Microbiology Service. The bacterial stocks used in the study were: *Staphilococcus aureus MSSA and MRSA, Klebsiella peumoniae ESBL, Escherichia coli, Escherichia coli ESBL, Enterococcus faecalis, Pseudomonas aeruginosa*. We have estimated 30 PLGs frozen at −40°, obtained from whole blood of voluntary donators screened according to blood donation rules by abstaining from using FANS, platelet average value of 300.000, haematocrit $< 42\%$, centrifuged to get plasma rich in platelets (1/2.000.000 μl-advia meter). The in vitro test provided to estimate preparation sterility and Leucocyte contents: Kirby-Bauer method on Muller Hinton Agar. Two plates were sown for each bacterial stock. In one of them we laid a small disk on which 10 μl of PLG and 2 μl of trombina were set up, in the second one we laid a small disk with 2 μl of trombina and the inhibition halo was estimated. In addition to this, for each stock the synergic answer of the PLG was estimated with Cefazoline, Teicoplanine, Gentamicine, by mowing Muller Hinton's plate with one of the stocks and by laying a disk with 10 μl of PLG and 2 μl of trombone and 6 mm disk with the chosen antibiotic.
**Results** In line with the latest data of literature the efficiency of PLG versus *Staphilococcus aureus MSSA* and *MRSA* and synergy with chosen prophylaxis is provided.
**Discussion** PLG could be a local therapy for some infections.
**Conclusions** In the bone defect treatment PLG could play a double role: both accelerator and immuno-modulator with antibacterial efficacy with interindependent proceeding.

### Genetic effects of trabecular titanium on human osteobalsts like cells (MG-63): an in vitro study

V. Sollazzo*[1], F. Carinci[2], A. Palmieri[3], F. Pezzetti[4], L. Massari[1]

[1]Clinica Ortopedica, Università di Ferrara (Ferrara, IT);
[2]Istituto di Chirurgia Maxillofacciale, Università di Ferrara (Ferrara, IT);
[3]Laboratorio Interdipartimentale di Chirurgia Maxillofacciale e Ortopedia, Università di Ferrara (Ferrara, IT);
[4]Istituto di Istologia ed Embriologia Generale, Università di Bologna (Bologna, IT)

**Introduction** Pure titanium and titanium alloys are materials widely used in orthopaedics and dental surgery because of their desirable mechanical properties, chemical stability, and biocompatibility. Although excellent clinical results have been shown with these materials, they have several inherent limitations (low volumetric porosity, relatively high modulus of elasticity and low frictional characteristics). To solve the limitations of these solid metals and to improve the characteristics of prosthetic implants, a new porous titanium (PT) biomaterial has been developed.
**Materials and methods** Here we are testing the effect of PT on osteoblast-like cells (MG63) cultured on disks of this new material by using DNA microarrays containing 20,000 genes.
**Results** We identified several genes covering a broad range of functional activities whose expression was significantly up or down-regulated. The data that came out from this study can give a detailed description of the genetic effects of porous titanium on human osteoblast like cells in vitro.
**Discussion** We demonstrated that PT seems to limit osteoclastogenesis and inflammation, main responsibles for the orthopaedic implants failure. Moreover, PT stimulating osteoblast proliferation and differentiation and reducing osteoblast apoptosis can favor the implant integration in the surrounding bone. At the same time PT, modulating genes related to immune system, seem to reduce the immunologic events against the prosthesis, therefore improving osseointegration and survival of the implant.
**Conclusions** These reported data encourage the clinical use of porous titanium implants.

### Biotechnologies with stem cells and growth factors: applications in cartilage traumatology

M. Leigheb*, M. Bosetti, M. Cannas, F.A. Grassi

SC Ortopedia-Traumatologia, AOU Maggiore D.C, Università del Piemonte Orientale (Novara, IT)

**Introduction** In the orthopaedic-traumatological field there is growing interest for biotechnology and tissue engineering evolution.

Growth factors (GF) are essential for endochondral bone growth and FGF-2 or TGFß2 overexpression was shown to enhance joint cartilage repair in vivo. Human mesenchimal stem cells (hMSCs) reveal the capability of generating bone tissue via an endochondral programme if implanted in vivo as engineered hypertrophic cartilaginous tissue, whereas to preserve cartilaginous characteristics the generated tissue needs to evidence no hypertrophy. Aim of the study is to evaluate the effect of different GF on hMSCs for applications in cartilage tissue engineering.

**Materials and methods** hMSC cultures were added with Fibroblast Growth Factors (FGF-2,-4,-6) and Transforming Growth Factor beta (TGFß). RT-PCR has been used to evaluate the expression of type II collagen and Sox9 as gene markers of chondrogenesys, while biochemical measurements and immunohistochemistry were used to evaluate GF's effects on extracellular matrix in 3D cultures.

**Results** Our findings, evidencing proliferating activity and differentiating potentials of FGF-2 and FGF-6 combined with TGFß2, suggest that they are powerful inducers of chondrogenesis in adult hMSCs, even though we have never reached the structure of adult hyaline cartilage.

**Discussion** We do not think we will be able to reproduce in vitro a tissue morphology similar to that obtained in vivo, because the system we used lacks some physiological factors important for cartilage maturation, such as mechanical loading.

**Conclusions** These studied GF combinations can provide a new tool to chondro-differentiate hMSC for applications in cartilage tissue engineering. Cartilage healing still represents a challenge in orthopaedic clinical practice but this basic science study might be useful to develop novel strategies in regenerative surgery.

### Biophysical stimulation in orthopaedics and traumatology

L. Massari*[1], M. Fini[2], M. De Mattei[3], K. Varani[4], S. Setti[5], R. Cadossi[5]

[1]Clinica Ortopedica dell'Università (Ferrara, IT);
[2]Istituti Ortopedici Rizzoli (Bologna, IT);
[3]Istituto di Istologia dell'Università (Ferrara, IT);
[4]Istituto di Farmacologia dell'Università (Ferrara, IT);
[5]Igea S.R.L. (Carpi-Modena, IT)

**Introduction** The most recent trends in orthopaedics and traumatology consider two different steps: accelerating the healing processes, particularly for the bone, and enhancing the functional status of the damaged tissues, especially in the cartilage. These objectives consider an increased anabolic activity of the tissues. There are different techniques that use engineering tissues, mesenchymal stem cells, cultured cells, local release of growth factors and BMPs in the site of the lesion. We also have to consider the possibility of intrinsical repairing and regeneration that can heal the damaged tissues in most cases.

**Materials and methods** Biophysical stimulation is a non-surgical technique capable to enhance and optimize the endogenous regenerative and healing possibility of the tissues. For example in cases of delayed union of bone fractures the biophysical stimulation is able to enhance the synthesis and the release of TGF-beta stimulating the endogenous production of bone morphogenetic growth factors. This work can explain the efficacy of the biophysical stimulation in traumatology, with an 84% of healing of non-union fractures. The physical stimulation using pulsed electromagnetic fields has an important role also in the condroprotection, in the repair of cartilage lesions, and in the integration of the engineering tissues for the treatment of condral lesions.

**Results** Two important arguments justified the use of pulsed electromagnetic fields in the treatment of lesions of the cartilage: (a) the ability to control the damage due to the pro-inflammatory citokynes (IL-1, TNF-alfa), as chondroprotective effect; (b) the stimulation of the synthesys of proteoglicans and the enhancement of the growth factors such as IGF-1 and TGF-beta. Biophysical stimulation significantly reduces the recovery in the treated patients in spite of placebo patients ($p < 0.01$). Furthermore, the 2- and 3-year follow-up studies demonstrated that patients treated with pulsed electromagnetic fields after surgical treatment of microfractures or chondrocyte transplants had a more rapid and prolonged recovery in spite of patients in the placebo groups.

**Conclusions** This positive effect can be explained with the global chondroprotective action of the biophysical stimulation over the cartilage. The biophysical stimulation is a non-invasive biotechnology that can favour the healing capacity of bone and cartilage.

### Surgical treatment of tibial plateau fractures with the addition of Cerament$^{TM}$ injectable bone substitute

E. Gasbarra*, D. Lecce, R. Iundusi, J. Baldi, A. Scialdoni, U. Tarantino

U.O.C. Ortopedia e Traumatologia, Fondazione Policlinico Tor Vergata, Università degli Studi di Roma "Tor Vergata" (Rome, IT)

**Introduction** The tibial plateau fractures involve more often young people as a result of high-energy traumas. When those fractures involve people over 50 years, they are most frequently due to low-energy traumas that highlight a condition of brittle bones. In either case, both the displacement and comminution of the fragments and the poor quality of bone may help the use of injectable bone substitutes associated with the use of adequate fixation. This study aims to assess the validity of the association of bone substitute Cerament$^{TM}$ to the surgical fixation for the treatment of tibial plateau fractures.

**Materials and methods** From February 2009 to February 2010, 16 patients with fractures of the tibial plateau were hospitalized in our Department of Orthopaedics and Traumatology (average 46.75 years; range, 32–64 years). According to the AO/OTA classification, 8 fractures of type B2, 5 fractures type B3, 1 type C1 and 2 type C3 were identified The patients, 8 males and 8 females, underwent surgery for reduction and fixation with fixed-angle plate (10 patients) or cannulated screws (6 patients) and augmentation with bone substitute Cerament$^{TM}$ (bone substitute ceramic material, consisting of injectable radiopaque calcium sulphate and hydroxyapatite) which promotes a more direct contact between implant and bone and acts as an osteoconductive matrix. The results were evaluated by using the clinical and radiographic Rasmussen scale.

**Results** Use of bone substitute Cerament$^{TM}$ ensured adequate filling of the defect determined by the tibial sinking and provided effective support to the surgical fixation for maintenance of stable reduction. Furthermore patients underwent an early mobilization of the limb the day after surgery and started the load on average 2–4 weeks after surgery.

**Discussion** The comminution and multifragmentation of tibial plateau fractures by high-energy traumas, as well as poor bone quality in the low-energy traumas, make the treatment very difficult. The treatment of these fractures requires the use of systems and strategies aimed both to restore a smooth articular surface stable under load and to recovery of motion as fast as possible.

**Conclusions** For the surgical treatment of tibial plateau fractures the use of CeramentTM augmentation in combination with an adeguate fixation, in our view, represents a valuable support to achieve satisfactory results in terms of maintaining the reduction, mobilization and weight bearing.

### Long-term results of MACI technique for the treatment of focal cartilage defects

R. Valentini*, P. Dordolin, S. Bernobi, G. Fancellu

Clinica Ortopedica e Traumatologica, Università degli Studi (Trieste, IT)

**Introduction** The articular cartilage lesions are produced by different mechanisms during trauma (by forces of compression and cutting, chronic instability, from outcomes of fractures). Whatever the type of trauma that causes the cartilage, lesion is always associated with an action due to aggravation of histopathological inflammatory phenomena. But we know that the articular cartilage defects heal with poor clinical results. Multiple surgical techniques have been proposed for the reconstruction of cartilage. We used the MACI technique in situations of focal cartilage degeneration. We wanted to evaluate the clinical and instrumental effects in patients after a long-term follow-up (up to 9 years).
**Materials and methods** We reviewed 20 patients (12 male and 8 female) with a mean age of 42 years (min. 23, max. 50), operated from January 2003 to May 2006 with MACI technique. The average diameter of the defect was 3.2 $cm^2$ (1.3–6.6 $cm^2$). All defects were classified according to Outerbridge grade IV. The follow-up period ranged from a minimum of 4 years to a maximum of 9 years. Evaluation eas performed by the Meyer score, the Lysholm score and the ICRS, and MRI control. In one patient we studied an arthroscopic removal specimen by electron microscopy examination.
**Results** Patients were subjectively very satisfied and returned to sports activities previously carried out. Significant increases in all the scores were seen up to 6 years after surgery. The unsatisfactory cases were those subsequent to fractures of the "tibial plateau". The MRI showed an evolution of the filling with normalization of the signal after 2 years and persistence of a hyperintense signal in 45% of cases.
**Discussion** The MACI technique in our cases showed good clinical results and optimal patient satisfaction. Cases with unsatisfactory results were those after "tibial plateau" fractures with a need for a wide coverage by the MACI membrane (4.5–6.6 $cm^2$).
**Conclusions** The MACI remains a possible technique for restoration of articular cartilage surface, able to obtain excellent long-term results in selected cases.

### *C41—BIOMATERIALS AND COATINGS IN ORTHOPAEDICS 1*

### Longevity® polyethylene in primary total hip arthroplasty: wear reduction at an average 7-year follow-up

M.F. Surace*, A. Fagetti, L. Monestier, D. Marcolli, P. Cherubino

Università dell'Insubria (Varese, IT)

**Introduction** In order to reduce the wear of bearing components in Total Hip Arthroplasty (THA), traditional UHMWPE acetabular liners have been replaced by Highly Crosslinked Polyethylene (XLPE) ones during last decades. This new material consists in covalent bonds that link one Polyethylene chain to another, with beneficial effects for highly increasing the long-term wear resistance and consequently for reducing complications as aseptic loosening.
**Materials and methods** Clinical and radiographic parameters of 54 patients who underwent THA (Trilogy® Acetabular System Tivanium® Ti6Al4 V cup—Zimmer, Warsaw, IN, USA; Versys® Hip System Zimaloy® Co–Cr–Mo femoral head—Zimmer, Warsaw, IN, USA; Versys® Hip System Tivanium® Ti6Al4 V femoral stem—Zimmer, Warsaw, IN, USA; Trilogy® Acetabular System Longevity® Cross-linked Polyethylene liner—Zimmer, Warsaw, IN, USA) due to primary or secondary coxarthrosis between January 2002 and December 2004.Clinical evaluation was based on Harris Hip Score, while radiological evaluation was obtained by the means of Roman software. Data were analyzed by means of statistical software SPSS 11.0.
**Results** Harris Hip Score significantly improved from pre-operative 52.77 to post-operative 89.65 points. Radiographic evaluation showed 0.070 mm wear during first post-operative year and 0.040 mm during the second post-operative year. Wear was 0.059 mm (0.011 mm/year) after first two years, when bedding-in phenomenon is widely considered concluded.
**Discussion** Present results confirm already published data for different types of XLPE, showing a significant wear reduction and consequent debris production, with optimal functional results and complications rate.
**Conclusions** XLPE really shows a high long-term wear resistance due to its chemical and physical properties; consequently, it can be considered as gold standard material for liners in THA.

### A new method for the creation of nanostructured anti-wear coating on the load surfaces of joint prosthesis: preliminary analysis

A. Russo*[1], N. Lopomo[1], A. Ortolani[1], C. Taliani[2], M. Marcacci[1]

[1]Laboratorio Nabi (Bologna, IT);
[2]Organic Spintronics Srl (Bologna, IT)

**Introduction** More than 40 years ago, ultra-high-molecular-weight polyethylene was introduced as the basic material for the development of loading surfaces in joint prosthesis. Nowadays it is the most common material used in prosthesis surgery and is still the limiting factor for long-term survival of the implant. Polyethylene debris, generated by continuos friction on the sliding surface, determine a cronic inflammatory reaction that leads to a progressive resorption of peri-prosthetic bone, to mobilization of metallic components of the prosthsis and, finally to failure of the prosthesis implant. The purpose of this study is to present anti-wear properties of hard nanostructured coatings made on the insert of polyethylene using a deposition system under high vacuum.
**Materials and methods** 3 samples (discs of 40 mm Ø×10 mm of thickness) of standard ultra-high-molecular-weight polyethylene were used as substrate for preliminary depositions. A ceramic target was used as material for the ant-wear coating realization; specifically the material from the target was deposited on the sample below, thereby integrating the substrate with a thin film of nano-structured coating. Nano-indentation preliminary test and tribology (coupling pin-on-disk with hemispheres of Steel 100Cr6 5 mm Ø) were performed on the deposited samples; an UHMWPE disc was used as control sample.
**Results** Depending on the parameters chosen for the deposition, the thickness of the thin film amounted to about 1–2 μm. Nano-structured coated samples presented an average hardness HIT of 467.98 ± 68.96 MPa (compared to 42.23 ± 0.83 MPa of UHMWPE), an average elastic modulus EIT of 1.96 ± 0.35 GPa (compared to 1.10 ± 0.02 GPa of UHMWPE) and an average wear rate WRate (2.37 ± 3.35)*10–5 g/(km*N; compared to 4.85*10–2 g/(km*N) of UHMWPE).

**Discussion** Results highlighted that is possible to create thin films of ceramic material on substrate of a plastic material particularly widespread in orthopaedic prosthetic couples (UHMWPE). Adhesion obtained by this new technology was very satisfactory from the qualitative point of view (uniformity and resistence of the film) also by varying the deposition parameters. Although roughness of the substrate played a particular criticality, preliminary characterization of obtained films has also demonstrated to be very encouraging, but now it's necessary to define quantitatively mechanical and tribological characteristics of the deposition, in particular identifying hardness, elasticity and scratch and mechanical wear resistance of the obtained material on a wider set of samples, thus varying typology of the substrate and of the coating material.

### Oxinium resurfacing implant artrhoplasty for earlier treatment of bicompartmental patello-femoral and medial femoro-tibial knee osteoarthritis

S. Zanasi*

Villa Erbosa Hospital, Gruppo San Donato (Bologna, IT)

**Introduction** In the past, the treatment of osteoarthritis of the knee was limited to implant unicompartmental or total knee arthroplasty. No option was ideal for active patients with medial and patella-femoral compartment osteoarthritis. A new monolithic two-compartment system is now available, in the philosophy of "Earlier intervention" for patella-femoral and medial compartment involvement, leaving intact the cruciate ligaments and the external compartment, thus maintaining the functionality of the almost normal knee.

**Materials and methods** 55 patients with concurrent arthrosis of the patella-femoral joint and medial tibio-femoral compartment were treated with the Deuce implant arthroplasty (Smith & Nephew) from June 2009 to December 2010: 21 patients were males and 34 females, aged 40–84 years (mean age, 56 years). In 4 patients with bilateral involvement, surgical procedure was performed bilaterally at the same time. All patients had pain, not severe limitation of ROM. The preoperative MRI showed neither damage of cartilage and meniscus of the lateral compartment, nor injury or LCA/LCP. The onset of symptoms dated from 1 to 4 years (mean 2.2 years).

**Results** The operative time was 55.0 ± 11.7 min. Blood loss of 280 (± 130) ml was clearly lower than in patients treated with TKA, and none required a blood transfusion. All incisions healed by first intention. Thirty-two patients were followed from 12 to 18 months (mean 14.2 months). Two weeks after surgery, most patients were able to walk only with a minimal limp and without the use of assistive devices. Patients had no patellofemoral pain nor clunking, no pain on palpation on the external compartment. There were highly statistically significant differences between preoperative and postoperative IKDC and KOOS scores: at 1, 3, 6, 12 months after surgery both clinical parameters and the ROM significantively improved (average post-operative ROM 0–134°). All patients reported a high level of satisfaction after implantation Deuce.

**Conclusions** The most important advantages of the two-compartment prosthesis coating are: (1) the preservation of the cruciate ligaments, which can satisfy the needs of normal kinematics and proprioception; (2) the minimally invasive surgical technique that is particularly suitable for this procedure and allows a quicker recovery to normal activity, (3) the increased stability and the decreased pain compared to the TKA. An important finding is related to the neutral axis correction in order to avoid the error of the correction in physiological valgus as in TKA.

### Unicompartmental knee arthroplasty: a comparative study between different tibial components

R. Valentini*, G. Fancellu

Clinica Ortopedica e Traumatologica, Università degli Studi (Trieste, IT)

**Introduction** We started the use of unicompartmental knee arthroplasty, believing that the replacement of the only pathological compartment with a small surgical access, sparing the ligamentous structures lead to more physiological results. To this aim we have choosed two types of unicompartmental prostheses, one with tibial component with a "metal back" and one with "all-poly" tibial component, in order to detect any differences between the two constructive solutions.

**Materials and methods** 50 patients were divided into two groups of 25 (*group A* "metal-back" and *group B* "all-poly"), homogeneous for sex and age (mean age, 68 years), operated from 2003 to 2009 and finally evaluated with a follow-up of 36 months. We used the Knee Society Score and radiographs of the knee under load for studying mechanical axis and joint line and the projection for the patella. Were evaluated the characteristics of body weight. All patients were operated with parapatellar medial access, without eversion of the patella and tourniquet and with early load.

**Results** The average KSS (objective and functional) pre-operatively was 48 for *group A* and 49 for *group B* and post-operative of 95 and 94 respectively for *Group A* and *group B*. The average postoperative ROM was 125° (85°–140°) of flexion–extension for *group A* and 130° (90°–145°) in *group B*. The alignment of the varus was found to be correct by about 5 degrees for both groups. At 5 years, the survival of the implants in *group A* was found to be 100%, while that of *group B* was 96%. We had no major complications with a discharge from the hospital in eighth days with a minimum of 2 and a maximum of 10 days of hospitalization.

**Discussion** We believe that the unicompartmental prosthesis is a useful indication for surgery in patients over 60 years with osteoarthritis of one compartment of the knee, with patellofemoral asymptomatic or mildly symptomatic, non-obese and non-rheumatoid arthritis or severe osteoporosis. We found no differences in the implantation of a tibial all poly, always greater than 6 mm thick, or of a "metal back".

**Conclusions** To achieve positive results over time, the accurate patient's selection and proper positioning of the components are very important. The operation is performed with little access and reduced surgical trauma, sparing the extensor and with rapid functional recovery and a social life with full outcome satisfaction for both the patient and the surgeon.

### Large diameter metal-on-metal in total hip replacement: are there indications for this type of implant yet?

A. Carfagni*, F. Clerici Bagozzi, F. D'Imperio, C.F. De Biase

Rome, IT

**Introduction** Use of metal metal coupling has long been criticized in recent years for the risks of systemic and local metallosis. It is important to note the low rate of complications compared to the risks described and the excellent clinical results. The question is: can this type of prosthesis still find space in modern surgery? We evaluated results of our experience and international literature.

**Materials and methods** More than 100 patients classified by age and sex were evaluated, we performed a clinical and radiographic follow-up 1–3–6–12 months and annually. The clinical evaluation was performed with the pre-operative Harris Hip Score at 6 months and over the years. Occurred complications were recorded and studied. Average follow-up was 23 months.

**Results** Radiographic examination showed a good implant integration in all cases, with a low rate of periprosthetic calcifications and no mobilizations. Results at 2 years were unchanged. This study shows a clinical improvement in ROM and in pain. Complications identified: two traumatic dislocations, 1 case of local metallosis.

**Discussion** This implant demonstrated a very satisfactory clinical picture. Both the radiographic and clinical follow-up showed a substantial long-term success of the system, with a good bone integration and a high rate of satisfaction in those patients over 65 with high functional demands. We believe that complications have to be linked to technical errors or traumatic outcomes. The presence of metal can be considered a contraindication for young patients. It is important to note that the pre-operative planing is essential in addressing choice of the right implant.

**Conclusions** We believe that in younger patients the risk is too high compared with the advantages, while it may be useful in patients over 65 with high functional demands. On the other hand, the new ceramics with large diameter heads could easily substitute metal heads. We, also, considered a risk, the metal degeneration of periarticular soft tissue.

### Use of ultra-high weight polyethylene added to vitamine in total knee arthroplasty

E. Bonicoli*, P. Parchi, I. Castellini, M. Lisanti

1[a] Clinica Ortopedica, Università di Pisa (Pisa, IT)

**Introduction** Periprosthetic osteolysis represents the most common cause of prosthesis revision; wear debris, primarily generated from polyethylene inserts, remains the major factor limiting the survival of joint implants. Polyethylene fragmentation and debris is due to a wide range of reasons including storage conditions, sterilization method, oxidation and, last surveyed, the molecular cross-link and the molecular stabilization obtained during the manufacturing process.

Recently the use of a cross-linked polyethylene added with vitamin E has been introduced in THA. However, it is well-known that a high cross-linking changes polyethylene's mechanical properties and makes it more rigid and brittle; for these reasons high cross-linked PE are less used in TKA.

**Materials and methods** Recently, a new cross-liked polyethylene added vitamin E (VITAL-E® Permedia) has been introduced in the market for the use in TKA. This is a high molecular weight polyethylene added with vitamin E as an antioxidants agent. In the VITAL-E® alpha-tocopherol is added to the polyethylene before extrusion (0.1% by weight) in order to give the material anti-oxidant properties without changing its mechanical properties.

**Results** The short follow-up does not express any information regarding the extent of the VITAL-E® polyethylene wear. We have not registered any complications on the plants achieved.

**Discussion** In the last years several solutions have been proposed to reduce polyethylene wear and research has been focused on using alpha-tocopherol (Vitamin E) as anti-oxidant agent in polyethylene. In all the areas where it is used, polyethylene is fortified with a stabilizing agent to prevent the material oxidation. Vitamin E is the natural mostly used worldwide stabilizing agent and it has been used for decades as an antioxidant agent for polyethylene thanks to its characteristics of bio-compatibility, availability and effectiveness.

**Conclusions** The aim of this study is to summarize the current causes of polyethylene wear and to report our preliminary impressions about the use of Vital-E polyethylene in TKA.

### *C42—BIOMATERIALS AND COATINGS IN ORTHOPAEDICS 2*

### Porous Titanium Regenerex® cup revision: clinical and radiographic results

E. Bonicoli*, S. Marchetti, M. Maltinti, L. Andreani, M. Lisanti

1[a] Clinica Ortopedica, Università di Pisa (Pisa, IT)

**Introduction** The Porous Titanium Regenerex® cup offers a primary stability during revision surgery due to its biocompatibility and a bone in growth from native bone. The roughness creates a high scratch-fit and at the same time, high mechanical strength. The structural biocompatibility allows the filling of pores that reaches 80% within 26 weeks from experimental data. It also allows an early load. The purpose of this study is to report our experience using the Biomet® Regenerex cup.

**Materials and methods** In the Orthopaedic Clinic of Pisa from September 2008 to January 2011, 20 patients underwent a revision surgery using Regenerex® cup and one patient underwent the first plant all with a mean age of 68 years, five males and 16 females, 13 with replacement also of the stem. The diagnosis was aseptic loosening in 19 patients, 1 septic. In 19 cases, screws were required for the low contact surface of living bone and metal. In 10 cases allograft was used made by 50% of bank bone and 50% TCP/HA. Preoperative evaluation included a study of clinical and radiographic hip, HHS and WOMAC scores. The surgery was performed by one operator with a postero-lateral approach. Radiographic evaluation was performed according to the ARA scores. The follow-up was 15 months.

**Results** In most cases the results were satisfactory with few/no symptoms (HHS > 83; WOMAC > 75). Neither lines of radiolucency or loosening were shown. The partial load was immediate with crutches and full load at about 60 days. As for complications, we recorded 2 cases of gluteus pain, 1 case of periprosthetic calcifications and 1 case of anterior pain from ileum-psoas impingment. Nothing related to Regenerex cup. Integrating X-ray was verified in all cases. The asymmetry was always less than 1.5 cm.

**Discussion** The Porous Titanium cup is a good choice in the revision because it offers a high roughness (roughness average 67%) that facilitates the primary stability allowing at the same time the bone in growth. Screws could be put in all directions and various augments can be used with 8 screw holes of 6.5 mm.

**Conclusions** Our experience with this type of cup is short, but the results are very satisfactory. From the preliminary results, this cup may be a solution in the revisions with a bone stock loss according to GIR 1 and 2 and some 3 GIR.

### Modular porous tantalum acetabular cup: clinical and radiological experience

M. Murena*, G. La Barbera, F. D'Angelo, M.F. Surace, D.A. Falvo, P. Cherubino

Varese, IT

**Introduction** Acetabular aseptic loosening is the main cause of failure in prosthetic hip surgery and the first cause of revision. Uncemented hemispherical cups are widely used and different biomaterials have been developed to obtain primary and secondary stability through osteointegration. Porous tantalum's structure is similar to cancellous bone and presents with mechanical and biological characteristics aimed to enable rapid and complete osteointegration and improve the survival of the prosthesis.
**Materials and methods** From May 2004 to June 2007 one hundred TMT modular acetabular components were implanted in 97 patients for primary THA. For this study we assessed 89 prostheses in 86 patients with 56 months (42–77 months) follow-up, both clinically and radiographically.
**Results** At the last follow-up no implant appeared mobilized. Harris Hip Score changed from 52.75 points preoperatively to 95.72 postoperatively ($p < 0.0001$). Oxford Hip Score improved from 37.16 to 14.78 points ($p < 0.0001$). Gaps at the bone-prosthesis interface were documented at the immediate postoperative X-rays in 51 implants (57%), mostly in zone II. At the last radiographic assessment they appeared completely filled in 87% of the cases (mean time to fill 1 year), partially filled in 10%, leaving less than 1 mm gap, while in 2 cases they remain unchanged. Osteointegration signs according to Moore and McAuley were also considered: at least three signs were present in all cases indicating complete osteointegration of the implants. There was no evidence of radiolucent lines at last follow-up. One dislocation occurred in one case at 21 months and it was reduced in narcosis with no recurrence.
**Discussion** Medium-term results of this cup are comparable with those reported in literature for porous tantalum monoblock components. The presence of the titanium layer in the metal-back of the modular component, that theoretically could affect the biomechanical characteristics of the cup, does not seem to affect host bone biological response when compared to the monoblock socket. Thanks to its mechanical characteristics and excellent osteoconduction, this biomaterial provides a good primary stability and excellent osteointegration of the component.
**Conclusions** The results reported suggest that this cup represents at a medium-term follow-up a good implant for acetabular arthroplasty.

### Tantalum metaphyseal cones in revision knee arthroplasty

V. Izzo*, V. De Santis, G. Milano, G. Logroscino, C. Fabbriciani

Università Cattolica del Sacro Cuore (Rome, IT)

**Introduction** The use of Tantalum cones (TMT) in revisions of total knee arthroplasty allows a better management of severe bone loss, which ranks as an alternative to allografts or tumor prosthesis. The best treatment of such bone defects is not yet well described in literature.
**Materials and methods** Thirty-two Tantalum cones, 19 tibial and 13 femoral were used in 45 revisions of total knee arthroplasty, 27 CCK-L®, 5 RHK®. Inclusion criteria were: IIb-III bone defects (AORI). The surgical technique consists in the placement of cones using the "3-point fixation technique". All patients were followed by clinical and radiographic evaluation for an average follow-up of 5.1 years (range, 4.2–7.4).
**Results** The HSS score increased from a pre-operative average value of 31 to a post-operative value of 88 (survival 94%). Radiographic study (Knee-Society) demonstrated in all cases signs of bony-ingrowth. No-progressive radiolucent lines were observed at bone-cones interface in 9 cases.
**Discussion** The clinical results reported in the literature, according to our study, demonstrate that Tantalum cones represent a true option in the treatment of severe bone defects. The use of "3-point fixation technique" showed satisfactory results at 5-year follow-up, but further analysis with longer follow-up and comparative studies are necessary to establish its efficacy.

### Revision of total hip arthroplasty after fracture of a ceramic component

G. Grano*, P. Lorenzon

UOA Ortopedia e Traumatologia, Ospedale Civile (Cittadella, IT)

**Introduction** Mechanical properties of the ceramic components in total hip arthroplasty markedly improved due to the progress in the process of manufacture. Nevertheless, fractures of any ceramic component are still encountered. The aim of this study is to examine the results of revision of total hip replacement performed specifically to treat a fracture of acetabular component or ceramic femoral head, and to identify technical factors that affected the outcome in short and medium terms.
**Materials and methods** In our Hospital, two surgeons performed 24 surgical revisions of total hip arthroplasties to treat a fracture of a ceramic component in the period 2000–2010. It was examined the clinical and radiographic documentation related to the time before the first arthroplasty and that related to the admission to the hospital for the revision. The patients were recalled and re-evaluated both clinically and radiologically; the radiographs of the last follow-up were compared with those of the post-operative time. The success of the revisions was evaluated by the survival analysis of Kaplan–Meier. The follow-up lasted from 3 months to 10 years.
**Results** The use of polyethylene acetabular components and ceramic heads with titanium sleeves, allowed us to obtain better results in short and medium terms, as regards to previously published reports. In fact we had no more precocious mobilization, ostheolysis and new fracture of the heads.
**Discussion** To obtain a good result, key factors seem to be the accurate removal of ceramic debris, a complete synoviectomy, and a gap as short as possible between the fracture and the revision. In our casuistry, fractures were more frequent in the acetabular side. Sometimes, fractures were connected with impingement mechanisms, due to the placement of the components, or to some eterotopic ossifications which caused extra-articulary impingement, or even (especially in young patients) to the utilization of the hip with a high ROM. Otherwise, we did not find mechanical causes.
**Conclusions** The fractures either of the head, or of the acetabular component, are exceptional complications; but they are dangerous.

An early diagnosis, with a fitted surgical treatment—i. e. an accurate synoviectomy, the removal of ceramic debris, and the use of ceramic/polyethylene interface—strongly reduces the short and medium term complications.

### *C43—BIOMATERIALS AND COATINGS IN ORTHOPAEDICS 3*

### Advantages of HA-coated screws in the treatment of intertrochanteric fragility fractures: surgical outcome evaluation in 160 patients

I. Cerocchi*, E. Gasbarra, A. Scialdoni, M. Celi, F.M. Liuni, F.L. Perrone, U. Tarantino

UOC Ortopedia e Traumatologia, Fondazione PTV, Università degli Studi Tor Vergata (Rome, IT)

**Introduction** In patients with skeletal fragility, the incidence of fracture-related complications increases, partly due to bone tissue characteristics and partly to inappropriate treatment choice. Aim of this study was to state if, in the treatment of intertrochanteric fragility fractures by intramedullary nailing, HA-coated cephalic screws can improve surgical outcome and accelerate functional regain, with respect to Titanium non-coated screws.
**Materials and methods** 160 subjects (120 females, 40 males) aged between 60 and 102 years (average age 81 years), hospitalized at our Division of Orthopedics and Traumatology due to intertrochanteric, low-energy trauma fracture, treated with Endovis BA intramedullary nail. All subjects had similar body mass index (BMI) and bone mineral density (BMD). 100 subjects were treated with the intramedullary nail and two HA-coated cephalic screws, and 60 with the nail and two non-coated cephalic screws. Patients were clinically evaluated immediately after intervention and after 1, 3 and 6 months using SF-36, test Timed Up and Go (TUG), VAS. Radiographic healing and the incidence of complications were also monitored.
**Results** No patient presented fracture-related complications. Subjects treated with coated screws showed faster functional regain, immediate weight-bearing and good TUG (24–30''), about 15–20 days earlier compared to non-coated screws. VAS documented faster pain reduction at the lower limb, and SF-36 a more rapid improvement of quality of life. Differences proved to be statistically significant between the two groups.
**Discussion** As documented by experimental and clinical studies, HA coating increases the holding power of bone implants, and can prove particularly useful when tissue biomechanics is compromised. The clinical results obtained, in terms of fracture healing and functional regain, were significantly better in subjects who received coated screws.
**Conclusions** HA-coated implants, in patients with poor bone quality can prove helpful in order to allow early weight bearing after a hip fracture, with better quality of life and reduction of the related local and systemic complications.

### Tribofit hip system in the surgical treatment of the medial femoral neck fractures in elderly patients

G.P. Molinari, V. Galmarini, R.M. Capelli*

SC Ortopedia e Traumatologia, A.O. Fatebenefratelli e Oftalmico (Milan, IT)

**Introduction** Tribofit Hip System (THS), consisting of a buffer made of a polycarbonate urethane plastic material, replaces the acetabular cartilage. Advantages using Tribofit Hip System are: minimal reaming and press-fit surgical technique. By ensuring that microelastohydrodynamic, hip lubrication is maintained, friction can be nearly eliminated, thus decreases the potential for wear. The aim of this clinical study is to evaluate a new acetabular buffer for elderly patients needing hip arthroplasty. We believe that optimizing the tribological fit at the synovial joint can improve outcomes and patient satisfaction.
**Materials and methods** Between 2008 and 2010 we recruited 24 patients at U.O. of Ortopedia e Traumatologia of Fatebenefratelli e Oftalmico Hospital of Milan, to evaluate the newly-developed THS. Inclusion criteria were: males and females between 70 and 80 years of age, femoral medial neck fractures requiring hip arthroplasty, body weight less than 100 kg., natural femoral head size. Patients were excluded on the following criteria: pathological fractures, leg paralysis, Parkinson disease, post TIA 1 year prior to surgery, severe concomitant pathologies. The mean age of the patients in this study (6 males, 18 females) was 76 years. All the patients were operated by the same surgeon. In all cases we used a cemented prosthesis stem (Symbios Arcad—Fin Bio—Impianti), bone caps and pressured cement (not antibioticated). Clinical evaluation was performed at pre-operative, post-operative, 1, 3, 6 and 12 months following surgery. Outcomes included: radiological assessment, Harris Hip Score, and ROM.
**Results** 9 patients completed a 12-month follow-up, only 6 patients completed a 6-month follow-up. Follow-up is still ongoing. All patients had no hip pain at 1-year follow-up; only three at 3-month follow-up had some hip pain (moderate). None of the patients had a dislocation, subluxation, or leg length discrepancy. We believe that the absence of dislocation is due to the relatively large femoral heads sizes (44–50 mm). None of the patients had sciatic paralysis, vascular accident or local infection.
**Discussion** The potential benefit of the Tribofit Hip System is minimal removal of acetabular bone which can considerably improve treatment in the elderly hip patients.
**Conclusions** Our preliminary results demonstrate the efficacy of the new Tribofit Hip System.

### Microstructural analysis of failed femoral stems revision in total hip replacement

L. Rizzi*[1], R. Chiesa[2], R. Maggi[1], C. Castelli[1]

[1]USC Ortopedia e Traumatologia, Ospedali Riuniti di Bergamo (Bergamo, IT);
[2]Dipartimento di Chimica, Materiali, Ingegneria Chimica, Politecnico di Milano (Milan, IT)

**Introduction** The failure of femoral stems in total hip replacement surgery is influenced by several factors that are related to the materials of the implants and the characteristics of the patient. In literature the reported incidence for the failure of femoral stems is variable between 0.23 and 11%, while very few papers that are related to the analysis of material for these implants are published. The aim of our study is to emphasize the importance of analysis of materials to determine the cause of the failure of revision femoral stem in total hip arthroplasty.
**Materials and methods** Three revision femoral stems, two cemented and one uncemented were explanted in our department because of failure due to fatigue fracture in two cases and in one case after bending deformation. All the stems were referred to the department of Chemistry of the Politecnico of Milan for investigation. Each component was examined with optical microscopy, SEM–EDS, metallographic analysis, microhardness tests and analysis of roughness for evaluating the microstructural characteristics and basic metals.
**Results** The rupture of the revision femoral stems was found in a cemented stem and a uncemented stem. In the case of cemented stem analysis performed showed a fatigue fracture occurred in a stem of austenitic steel with properties which do not correspond to the current ISO and with a diameter reduced in size. The uncemented stem of titanium has suffered a fatigue fracture by fretting corrosion, phenomena typical of this material. Finally, in the stem with bending deformation metallographic analysis showed a higher grain size and michrohardness values lower than the standard ISO indicative of a low rate of hardening of steel.
**Discussion** In total hip replacement surgery, especially in revision surgery, the materials of the femoral stem are important in determining a possible failure. The results of our study are able to detect as the mode of failure observed is related not only to the diameter, but also at the microstructural characteristics of materials used in processing.
**Conclusions** In revision total hip arthroplasty surgery where the anatomical conditions of the femur appear often compromised by the effects of aseptic loosening, the implants are subjected to increased stress load and the material characteristics of such implants should provide a greater margin of safety in order to avoid premature failure.

### New guide for the positioning of endomedullary nail in the combined femur lengthening

E. Astarita*, A. Monegal, J. Tapiolas, V. Bellotti, I.M. Ginebreda

Instituto Universitario Dexeus (Barcellona, Spain)

**Introduction** The techniques of femur lengthening have suffered in the last years an important evolution due to the use of endomedullary systems combined with external fixation. A new guide was disegned (in use with the TRIAX external fixators and STRIKER T2 femoral medullary nails) that allow an absolute safety of the proximal pins insertion, preventing the accidentally nail contact and decreasing the use of the X-rays.
**Materials and methods** We performed 22 femur endomedullary lengthnings in patients with inferior limbs dysmetria caused by different etiology.
**Results** We considered different parameters such as: the lengthening expectations, the median time of consolidation, surgical times and exposure to X-rays, days of external fixation/cm of elongation, total days of treatment/cm of elongation, complications, days need to rich consolidations/cm of elongation.
**Discussion** First of all, this guide avoids the contact among the components, thus decreasing the risk of contaminations. In second place, it decreases friction and wear risks of the components during the lengthening procedure that could affect the device stability at long term.
**Conclusions** This new guide allows proximals pins in absolute safety, preventing the accidental contact with the endomedullary nail and it decreases the use of X-rays.

### Total shoulder arthroplasty in primary osteoarthritis: long-term results

G. Merolla[1], G. Nastrucci*[2], G. Porcellini[1]

[1]U.O Chirurgia Spalla e Gomito, Azienda AUSL Rimini (Cattolica, IT);
[2]Servizio di Ortopedia, Campolongo Hospital (Campolongo-SA, IT)

**Introduction** The glenohumeral arthritis occurs in approximately 30% of the population aged over 60 years. Total shoulder prosthesis is the "gold standard" in patients with severe symptomatic osteoarthritis. The surgeon may use modular prosthetic systems widely adaptable to different conditions of shoulder joint. In this paper we review the surgical technique and report the long-term results in cases treated with total shoulder arthroplasty with a cemented polyethylene glenoid component.
**Materials and methods** Between January 2000 and December 2001 at our Center 158 total arthroplasties of the shoulder were performed. In the current study, we enrolled 30 cases. Overall patients were clinically evaluated with the Constant-Murley (CS) scale by comparing pre and postoperative values. The radiographic analysis was performed in the AP and axillary view made at the last follow-up to an average of 10 years (range, 09–11 years). The Student-*t* test was applied for the analysis of data collected with significance at 5% ($p < 0.05$).
**Results** The CS increased from an average of 21.4 (SD, 5.6) in the pre-operative to 62.6 (SD, 4.8) post-operatively ($p < 0.05$). In patients with pre-operative age lesser than 50 years the increase of the post-operative average values of the CS and ROM was greater than the sample mean. Periglenoid radiolucency was found in 20 cases (70%) with lines lesser than 1.5 mm, resulting in a thickness exceeding 1.5 mm in only 3 cases (10%). Perihumeral radiolucency was observed in 9 patients (30%), with incomplete lines of 2 mm in 2 cases (6.7%). Complete glenoid loosening was found in 2 cases (6.7%).
**Discussion** The clinical and radiographic outcomes of this study are consistent with other mid- and long-term follow-up studies.
**Conclusions** Anatomical total shoulder arthroplasty is an effective long-term treatment of severe glenohumeral osteoarthritis.
**Suggested reading**

1. Merolla G, Campi F, Paladini P, Porcellini G (2008) Efficacy of anatomical prostheses in primary glenohumeral osteoarthritis. Chir Organi Mov 91:109–115.

### Trabecular Titanium™ associated with a hydrogel enriched with osteoinductive factors and bone marrow stromal cells: an innovative strategy to ameliorate prosthetic implants

S. Lopa*[1], S. Pedroli[1], M. Tavola[1], L. Zagra[1], F. Segatti[2], G. De Conti[3], D. Mercuri[3], M. Moretti[1]

[1]Laboratorio di Ingegneria Cellulare e Tissutale, IRCCS Istituto Ortopedico Galeazzi (Milan, IT);
[2]Lima Corporate Spa (Villanova di San Daniele, IT);
[3]Biosuma Srl (Villanova di San Daniele, IT)

**Introduction** Trabecular Titanium™ (TT) is a novel material with a structure similar to trabecular bone, already used for prosthetic clinical applications. Being the bone-implant interface the weakest point during healing process, the association of TT with a hydrogel enriched with cells and osteoinductive factors may represent a promising strategy to improve osteointegration. In this study we evaluated viability and osteogenic differentiation of human mesenchymal stem cells from bone marrow (BMSCs) cultured on TT combined with an ammidated carboxymethylcellulose hydrogel (CMCA). The effect of strontium chloride ($SrCl_2$) on BMSCs osteogenic differentiation was evaluated with the final aim of employing it to enrich TT-CMCA composites.

**Materials and methods** BMSCs were isolated from 7 donors and seeded on CMCA and on normal or CMCA enriched (TT-CMCA) TT disks (ø 8 mm, thickness 3 mm, pore size 640 μm). Biomaterials were individually tested for osteoconductivity using BMSCs cultured in osteogenic medium either in the absence or in the presence of 5 μg/ml $SrCl_2$. After 14 and 21 days, viability and osteogenic differentiation were evaluated by Alamar Blue assay and Alkaline Phosphatase (ALP) quantification. After 21 days the production of extracellular matrix was determined by Scanning Electron Microscopy (SEM).

**Results** BMSCs colonized CMCA and TT constructs, showing a higher viability when cultured on TT and TT-CMCA with respect to CMCA. Enrichment with CMCA improved the osteoconductive properties of TT, as demonstrated by ALP increase in BMSCs cultured for 21 days on TT-CMCA in comparison to TT alone (+42%). SrCl2 induced relevant increases in ALP levels in BMSCs cultured on CMCA, TT and TT-CMCA in respect with cells on the same materials in normal osteogenic medium (+313%, +352%, +264% after 21 days). In TT and TT-CMCA samples cultured in the presence of SrCl2, SEM analysis revealed abundant extracellular matrix, characterized by fibrillar structures.

**Discussion** TT and TT-CMCA well supported BMSCs growth and osteogenic differentiation. The enrichment of TT with CMCA allows the maintenance of BMSCs within the construct, which is particularly important considering a future clinical application where cells will be isolated and seeded on TT implants during a one-step surgical procedure. Furthermore, CMCA permits the inclusion of SrCl2 that may induce both the osteogenic differentiation of BMSCs seeded in the construct and of resident BMSCs recruited to the lesion site.

**Conclusions** Our results demonstrate that composite prosthetic devices made from TT and CMCA can be enriched with autologous BMSCs and SrCl2 and may represent a valid strategy to promote and improve implant osteointegration.

### Metal on metal tribology in total hip prosthesis: short-term results of Magnum acetabular cup

G. Grappiolo*[1], G. Cusmà[1], F. Astore[1], G. Mazziotta[1], E. Caldarella[1], A. Lupi[2]

[1]Istituto Clinico Humanitas (Rozzano, Milano, IT);
[2]Scuola di Specialità in Ortopedia e Traumatologia (Parma, IT)

**Introduction** Magnum M2A acetabular cup (Biomet) is an emisferical cup made of chromo-cobalt steel with metal on metal tribology. We collected clinical and radiographical data of our cases in a prospective study.

**Materials and methods** From December 2008 to December 2010 we implanted 247 Magnum cups with our standard technique (pre-op planning, postero-lateral approach, “Femur first”, underreaming by 1 mm) in 230 patients of which 216 males. The mean age at operation was 50.76 years (range, 20.7–85.9). The measures were the most commonly used 50 mm (59 cases), 52 mm (63 cases) and 54 mm (55 cases). These cups were implanted (in patients with no history of metal allergies) in combination with uncemented stems, chosen according to age, bone quality and morphotype: 61 CLS, 6 Conus, 97 Fitmore, 8 Metha, 5 Nanos, 70 GTS. Each patient performed a standard prophylaxis for infections and DVT; the hospital rehabilitation programme was between 10 and 16 days. All patients have a clinical and radiological follow-up of 45 days, 3, 6 months and 1 year after surgery: 137 patients have more than 12 months follow-up.

**Results** We did not have mechanical intra-operative complication, due to the instrument or system. Rare post-operative complications: 1 wound superficial infection treated with debridement, 1 aseptic loosening secondary to femur impingement without enough offset. We didn’t found areas of radiolucency around the cup, but a gap in the region of 2-Deleey Charnley in 5% of cases with a tendency to decline in subsequent checks.

**Discussion** Our experience with the Magnum cup is positive, with clinical and radiographic results of which there are overlapping, with the same follow-up to those previously observed by us for a similar cup (Zimmer Durom) implanted in large numbers until beginning of this series. Compared to our previous trend, there is a significant reduction of female patients in this series.

**Conclusions** The position of the cup is important for best results. We hope to have good results at medium and long term follow-up using the technique of “Femur first”, introduced at beginning of this study that permit to manage better the reciprocal position of the femur and cup.

### *C44—BIOMATERIALS AND COATINGS IN ORTHOPAEDICS 4*

### Acetabular revision with omnia modular system: a 250 case-review

G. Zanotti*, M. Pagaelli

Azienda USL di Ravenna (Lugo di Romagna, IT)

**Introduction** OMNIA system introduces innovative concepts in the field of acetabular prosthetic revisions: the aim is to provide the surgeon with various options for fixation of the acetabulum so that these can be implemented together as required depending on the seat and the severity of the bone defects encountered during surgery, as well as according to the operator’s personal preferences. Introduced modularity features simplify the surgical technique allowing for changes in the inclination and the acetabular rotation subsequent to cup implantation.

**Materials and methods** We present the results referring to 250 implant cases in the last 5 years.

**Discussion** The system can employ various types of fixation that can be implemented in different ways, with one common feature, i.e. mechanical principles of angular stability. The cup is hemispherical and three different fixation methods are possible; moreover it can be used alone or in association with one another one and the selection of the type and implementation of the fixation is done during the surgery on the basis of the complexity of the acetabular defects.

1. *SCREWS*: characterized by their large diameter (8.5 mm) and the device for angular locking in relation to the cup
2. *PEGS*: these are nails with six fins and tapered tip; they are inserted directly inside like the screws, after positioning the acetabular cup. In this case too, angular stabilization of the “peg” follows by means of a screw for locking to the cup.
3. *STEMS (ILIAC STEMS)*: are the “greater” fixtures of the systems, used in more complicated cases; they are joined to the outer surface of the acetabular cup by means of a screwed intermediate element and a special locking cap.

The possibility of transformation of the implant from a simple “press-fit” hemispherical cup to a system with multiple fixation aids made it possible to handle all types of acetabular prosthesis with a single system.
**Conclusions** The method makes it possible to easily handle unexpected intraoperative situations such as bone defects more serious than those encountered during pre-operative investigations, and it is therefore a suitable solution for all cases of acetabular revision.

### Clinical and densitometric preliminar results on osseointegration around acetabular cups with Trabecular Titanium

L. Massari[1], P. Rossi[2], M. Crova[2], A. Causero*[3], G. Gigliofiorito[1], P. Grillo[2], A. Bistolfi[2], C. Pari[1], C. Concina[3], P.M. Tosco[3], S. Burelli[4], H. Bloch[4], G. Carli[4]

[1]Clinica Ortopedica dell'Università (Ferrara, IT);
[2]Clinica Ortopedica dell'Università (Torino, IT);
[3]Clinica Ortopedica dell'Università (Udine, IT);
[4]Lima Corporate (S. Daniele-Udine, IT)

**Introduction** Trabecular TitaniumTM is a tri-dimensional material composed by multi-planar regular hexagonal cells and characterised by a highly open porosity that has been studied to optimise bone osteointegration. The aim of this study is to evaluate bone remodelling measuring BMD changes around an acetabular cup made from Trabecular TitaniumTM in primary total hip arthroplasty (THA).
**Materials and methods** Between February 2009 and December 2010, 89 patients (91 hip) underwent primary THA with a modular acetabular cup in Trabecular TitaniumTM (DELTA-TT cup, Limacorporate, Villanova di San Daniele, Italy). The average age was 63.5 ± 9.4 years, the average height and weight were 75.9 ± 12.9 kg and 168.8 ± 8.9 cm, respectively (average BMI 26.8 ± 4.2). There were 46 (51.7%) males and 43 (48.3%) females affected by primary coxarthrosis in 80 (87.9%) cases, avascular necrosis in 5 (5.5%), post-traumatic coxarthrosis in 3 (3.3%), dysplasia in 2 (2.2%) and oligoarthritis in 1 (1.1%) case. The study includes the clinical evaluation with Harris Hip Score (HHS) and SF-36, radiographic evaluation and dual-energy X-ray absorptiometry (DEXA) analysis pre-operatively and post-operatively at 1 week, 3, 6, 12 and 24 months.
**Results** Preliminary results are currently available for 47 patients at 12 months, 68 at 6 months and 80 at 3 months. The average HHS significantly improved from 48.7 ± 14.99 preoperatively to 93.8 ± 5.91 at 12 months, with a constant progression in the intermediate follow-ups. All patients showed a significant ROM increase, with an average flexion from 86.6° ± 15.9° preoperatively to 105° ± 13.14° at 12 months. Sf-36 highlighted a satisfactory improvement of general health status from an average preoperative value of 50.8 ± 18.7 to 80.7 ± 12.9 at 12 months (from 42.9 to 80.1 for physical health; from 58.4 to 81.3 for mental one). All cups were stable at 12 months with no radiolucent lines. Preliminary DXA analysis reported an initial bone mineral density decrease from 1 week baseline values (BMD R1: 1.40 ± 0.37; R2: 1.20 ± 0.45; R3:1.16 ± 0.31) to 3 months (BMD R1: 1.31 ± 0.41; R2: 1.17 ± 0.3; R3: 1.06 ± 0.37) followed by BMD recovery up to initial values (BMD R1: 1.37 ± 0.3; R2:1.18 ± 0.34; R3: 1.12 ± 0.36) at 12 months.
**Conclusions** Trabecular TitaniumTM demonstrates a good primary and secondary stability. Preliminary densitometric outcome confirms an optimal osseointegration of the DELTA-TT cup and early clinical and patient subjective results are very promising at a short term follow-up. However, the completions of follow-up evaluation are necessary to draw a conclusive analysis.

### Trabecular metal modular cup in major acetabular revision surgery

L. Pierannunzii*, A. Guarino, M. d'Imporzano

Istituto Ortopedico G. Pini (Milan, IT)

**Introduction** Anti-protrusio cages, stemmed cups, modular cups provided with obturatory hook and iliac flanges are widely accepted devices for major acetabular revision (GIR stage III and IV). Recently favorable series were reported with elliptical modular cups made of Trabecular Metal. Should this indication be confirmed by sounder evidence, it might simplify the surgical procedure significantly.
**Materials and methods** All hip revisions performed from 2008 to 2009 with implantation of a TMT multi-hole acetabular cup without augmentations were retrospectively reviewed. The cases with GIR stage I and II bone loss, with surgical report poorly describing the bone defect, with inadequate pre- and post-operative X-rays were ruled out. Twenty-five cases were identified, but four were lost to follow-up. The twenty-one patients were 71 year-old on average, with stage IV bone loss in 6 cases and stage III bone loss in 15 cases. Mean interval from surgery to evaluation was 20.9 months (from 13 to 30). The evaluation included bone-prosthesis contact estimation, survivorship, complications, final Harris Hip Score.
**Results** Mean host bone-prosthesis contact was estimated to be about 35%. Only three implants were subsequently reoperated (for infection, early migration, recurrent dislocation). The HHS among non-reoperated 18 patients was 81.96 on average (from 63.44 to 95.82).
**Discussion** The results are consistent with recent literature about major acetabular revision. Implanting a quasi-primary cup does not seem to be associated with higher failure risk than with more invasive, bluky, dedicated devices. The tendency towards center of rotation elevation did not affect the clinical outcome significantly.
**Conclusions** The mid-term results of this series confirm the hypothesis that a porous tantalum acetabular cup is an effective option to deal with major acetabular revisions, even without augmentations.

### Before talking of coatings, what really is the surface of orthopaedic implant devices?

M. Morra*, C. Cassinelli, G. Cascardo, D. Bollati

Nobil Bio Ricerche (Portacomaro-AT, IT)

**Introduction** Coating of orthopaedic implant devices is a widely investigated topic of great scientific and clinical relevance. As a part of our ongoing effort on bioactive coatings design, we performed some surface characterization work on implant devices from different producers. The study involved osteosynthesis screws, shoulder and knee prosthesis, vertebral fusion cages, dental implant fixtures. The aim of the work was the definition of a starting point for a rationale surface science of bone implant devices.
**Materials and methods** All test were performed on devices sealed and sterile, i.e. in the ready-to-use condition. Analysis involved scanning electron microscopy (SEM), and X-ray Photoelectron Spectroscopy (XPS) for the evaluation of surface chemistry. Adherent endotoxin was evaluated through a recently validated test involving RT-PCR measurement of gene expression by inflammatory cells (macrophages).
**Results** Obtained results show a rather complex picture, as follows: (1) prosthesis roughened by sandblasting show marked presence of residuals of the blasting medium; (2) most of the analysed devices show a surface chemistry deeply affected by process and cleaning residuals; (3) the amount of adherent endotoxin on devices surfaces is very variable, despite this parameter being controlled by standards. Some ostheosynthesis screw is more proinflammatory, because of adherent endotoxin, than our positive control, a sample immersed overnight in endotoxin solution.

**Discussion** Osteoimmunology, the study of the interaction between cells of the immune and skeletal systems, shows that a number of pathologies involving bone resorption, including peri-implant pathologies, are secondary to stimulation of osteoclastogenesis by inflammatory cytochines. Tests performed show that surfaces of most analysed implant devices contains significant inflammatory stimuli, both due to process residuals (chemical cleanliness) and to adherent endotoxin (biological cleanliness). This aspect is particularly important, because pathologies involving peri-implant bone resorption, such as aseptic loosening, generally considered caused by fragments and residuals from phrostesis, have recently been interpreted in terms of inflammatory response to adherent endotoxin on said residuals an fragments.

**Conclusions** Surfaces of analysed devices, all of them in current clinical use and compliant with current rules and standards, clearly show ample room for improvement, if considered from the point of view of biomaterials surface science. The inclusion of criteria to optimize devices surfaces-cell interaction, in particular with respect to inflammatory cells, should be part of every device design and production process, as a precondition to implementation of specific coatings.

### Modular megaprosthesis in prosthetic loosening with bone loss and in post-traumatic meta-epiphyseal defects or deformities

D.A. Campanacci*, N. Mondanelli, L. Ciampalini, P. De Biase, G. Beltrami, P. Cuomo, G. Scoccianti, D. Matera, M. Ippolito, R. Capanna

A.O.U. Careggi (Florence, IT)

**Introduction** Severe periarticular bone-loss may be post-traumatic or secondary to prosthetic failure, due to wear osteolysis, stress-shielding, infective sequelae and periprosthetic fractures. Rehiterative surgeries may furtherly jeopardize the residual bone-stock. Among surgical options, there are new attempts at osteosynthesis, revision with diaphyseal-fitting prostheses, impaction-grafting technique and the use of modular tumor prostheses. Such megaprostheses were designed for oncological reconstructions and showed good long-term results, but there are few studies that report the results in non-oncological diseases.

**Materials and methods** Between 2001 and 2010, 50 patients were treated with resection and megaprosthesis replacement for prosthetic failure (27 cases) or periarticular nonunion (23 cases), using the MegasystemC® (Waldemar Link®, Hamburg, Germany). Proximal femur was replaced in 24 cases (23 megaprostheses and 1 allograft-prosthesis composite, APC), distal femur in 16 cases, and total femur in 2 cases. In 1 case distal femur was replaced by a megaprosthesis and proximal tibia by an APC. Four patients underwent a knee arthrodesis with modular prosthesis. Proximal tibia was replaced in 3 cases (2 megaprostheses and 1 APC). In 5 cases with infective sequelae, MegasystemC with silver surface (PorAg) was implanted (2 proximal femora, 1 distal femur, 2 arthrodeses).

**Results** Ten major complications were observed (20%). Hip dislocation occurred in 3 cases (11% of hip reconstructions); 1 patient was treated with closed reduction while surgical revision with self-retaining acetabular component was required in 2 cases. Deep infection was observed in 2 cases (4%), healed after revision in 1 case and requiring hip disarticulation in the other. Two patients (4%) presented a periprosthetic fracture requiring surgical revision. One patient presented a wound dehiscence, with negative microbiological cultures, treated with surgical debridement, prosthetic substitution and rotational flap closure. One patient had a patellar tendon detachment treated conservatively. One case of persistent peroneal nerve palsy was observed. At an average follow-up of 16 months, in 34 evaluable cases, functional results according to MSTS classification were excellent in 9 cases, good in 13, fair in 9 and poor in 3 patients.

**Conclusions** MegasystemC® can be used successfully for reconstruction of severe bone-loss in prosthetic revision surgery and post-traumatic sequelae. In the elderly and osteoporotic patient, resection and prosthetic replacement can be considered as an alternative to revision with conventional implants or further attempts of osteosynthesis. Reduction of surgical time and blood loss and faster functional recovery with early weight bearing are major advantages of megaprosthetic reconstruction. Megaprostehses with silver surface can be an option in septic revisions.

### Total joint substitution in the treatment of hallux rigidus: our experience

R. Valentini*

Clinica Ortopedica e Traumatologica, Università degli Studi (Trieste, IT)

**Introduction** The degenerative joint disease of hallux rigid in the advanced stage has always been a challenge in the surgical treatment. Over the years there have been several proposals of joint replacement surgical techniques in order to relieve pain, correct deformities and to maintain a certain degree of motion. In our surgical experience, we used the first hinge osteotomy proposed by Valenti and then the replacement technique of the first metatarsal head with a bioresorbable spacer with the aim of creating a biological arthroplasty. With this technique we have shown consistently positive results in regard to the satisfaction of patients with increased range of motion, but with the appearance, albeit sporadic, of an important inflammatory foreign body reaction.

In recent years, for the treatment of severe hallux rigid (grade III and IV), we used a metal prosthesis (Reflexion).

**Materials and methods** We reviewed 20 patients (16 female, 4 male) with a mean age of 58.1 years, operated with this technique from June 2008 to June 2010. In all patients trunkal ankle anaesthesia was performed and granted immediate loading. Patients were monitored clinically and with radiographs with follow-up period of 18 months (min. 6 months, max. 30 months). Functionality was assessed by the score of Kitaoka et al.

**Results** The return to normal activities was around 28 days (min. 21, max. 65). The average score second Kitaoka had increased to 75 points at 3 months postoperative and 87.1 points after 6 months after surgery, starting with a preoperative score of 36.6. The reached average ROM was 42° (extension 25.3° and flexion 18.1°). We found no major complications or no loosening of the implants.

**Discussion** Over the years various techniques have been proposed to alleviate pain, restore and maintain the motility of the first MP in patients with hallux rigid. Our results appear to be in accord with those obtained by other authors using the prosthetic replacement in treatment hallux rigid. If these results were maintained over time, we believe that the prosthetic replacement will be a better surgical proposal than arthrodesis.

**Conclusions** Results seem to be favorable and the patients' satisfaction is complete. We believe that with this technique remains a degree of motion ensuring a good functioning of the forefoot and that solves the problem of pain while maintaining joint stability.

### Ten years experience (1999–2010) with LCS cementless TKA: bone-metal osteointegration and implants survivorship

M. Merlo*, A. Mazzucco, V. Occhipinti

Ospedale di Circolo (Busto Arsizio, IT)

**Introduction** We analyzed the results of TKA implants performed from 1999 to 2010 at the Busto Arsizio General Hospital, Varese, Italy using the LCS uncemented TKA (Low Contact Stress, De Puy, Warsaw-In), with PCL sacrifice and rotating platform.
**Materials and methods** The study was performed on over 700 implants, with large percentage of primary OA and a small number for secondary post surgical, post traumatic OA and RA. Our patella management consists in no resurfacing but shape and deformities remodelling and circumferential rim cautery for partial denervation. Our attention was oriented to evaluate bone—Porocoat interface with X-rays compared to the clinical evidences. Implants percentage of survivorship was evaluated, the mechanical failure (PMWWE, spin-off and dislocation) and septic loosening were analyzed and classified.
**Results** Results regarding the group with the longer follow-up demonstrated 97% of survivorship of the implants. No case of aseptic loosening of the prosthetic components of the TKA was found.
**Discussion** The collected data on a large number of cases in the 10 years (1999–2010) confirm the positive experience about the implants with Porocoat surface and its osteointegration.
**Conclusions** We reported that the Porocoat-bone osteointegration, associated to a correct surgical procedure, offers good fixation of the femoral component and also, not always predictable, of the tibial component.

---

### *C45—KNEE 3*

### Lateral release in navigated and conventional total knee arthroplasty

A. Della Rocca*, F.R. Ripani, P. Sessa, W. Salustri, G. Cinotti

Clinica Ortopedica, Università "Sapienza" (Rome, IT)

**Introduction** An appropriate patellar tracking is considered to be one of the key factors in order to obtain a satisfactory result after total knee arthroplasty (TKA). To accomplish this goal it is mandatory that the femoral component be positioned with a proper alignment, avoiding, in particular, an internal rotation of the prosthesis. If at the end of surgery an inadequate patellar tracking is found, a lateral retinacular release should be performed. However, such a surgical step increases bleeding and postoperative pain. Aim of the present study was to assess whether the need to perform a lateral release at the end of TKA occurs with similar rates after navigated or conventional TKA.
**Materials and methods** We prospectively evaluated 120 patients submitted to navigated (60 patients) and conventional (60 patients) TKA. Mean age was 69 and 71 years, respectively. All patients had a varus knee with a similar preoperative deformity in the two groups. In all cases a standard medial approach was used along with lateral patellar eversion. In the navigated TKA group the axial rotation of the femoral component was oriented averaging the Whiteside line, posterior condyles and epicondylar axis. In the conventional TKA group, the posterior condyles were taken as reference (plus 3° of external rotation). At the end of surgery, the patellar tracking was assessed during the whole ROM and a lateral release performed if needed.
**Results** In the navigated group the posterior condylar axis was internally rotated more than 3° compared to the Whiteside line in 38 cases (63%), and less than 3° in 3 cases (5%). A lateral release was needed in 4 (7%) of the patients in the navigated group and in 14 (23%) of the conventional group ($p < 0.05$).
**Conclusions** The results of this study indicate that the concomitant use of different anatomic landmarks, which can be accomplish with navigation, allows the surgeon to achieve a correct rotational alignment of the femoral component in a significantly higher percentage of cases than when the posterior femoral condyles only are used as anatomic landmarks.

### Rotational alignment of distal femur in computer assisted total knee replacement (TKR)

A. Todesca*, J. Bejui Hugues, M. Penna

Istituto Ortopedico Chirurgico e Traumatologico I.C.O.T. (Latina, IT)

**Introduction** Rotational alignment of the distal femur in TKR is usually referred to the bone landmarks in the conventional measured resection technique (femur first) or to the flexion–extension gap in the gap balancing technique (tibia first). Both techniques could lead to undesirable rotation of the femoral component, especially in knees with significant pre-operative leg axis deviation, causing patella maltracking and joint instability in particular in flexion. Using computer assisted surgery (CAS) rotational alignment may be archived combining data related to the bone morphing of the distal femur to flexion gap balancing data. Aim of this study was to examine the influence of the pre-operative leg axis on the rotation of the femoral component and the symmetry of the flexion gap after TKR using CAS.
**Materials and methods** In consecutive 144 patients pre-operative lower limb alignment was established in full weight bearing X-ray while femoral component rotation, varus-valgus laxities at 0° and 90° degrees of flexion were measured intra-operatively using the computer navigation data (Amplivision–Amplitude). The outlier of soft tissue balancing was defined as a gap difference >3 mm between the medial and lateral sides.
**Results** A mean external rotation of the femoral component of 1.5° (−4°/+ 6°) was achieved with a positive correlation of the preoperative leg axis with the rotation of the femoral component. Medial or lateral outliers in extension or flexion were observed in 12% of TKR. An intraoperative lateral patellar retinaculum release was performed in only two patients.
**Conclusions** CAS in TKR allowed not only to reduce the post-operative alignment outlier, but also to determine the optimal rotational alignment of the femoral component realizing well balanced rectangular flexion and extension gaps, optimizing joint stability in all the range of motion especially in more severe varus/valgus knees. Moreover an effective patello-femoral alignment may be easier achieved.

### Clinical audit and computer assisted surgery

F. Boniforti*, F. Giacco, F. Giangrasso

Fondazione San Raffaele Giglio (Cefalù, IT)

**Introduction** The reduction in wrong positioning and sizing implant is one of the most important aim of the computer-assisted surgery. In clinical practice, audit refers to a method of evaluation on the application and effect of new procedures. The purpose of the study was to apply an audit methodology to measure the effect of computer assisted surgery in knee prosthetic surgery in our hospital.

**Materials and methods** Our clinical practice is based on an activity of about 15 prosthetic knee surgeries a month for over 5 years. In this practice, we have introduced the procedure with surgical navigation and measured the changes and the approval of the new method. In two consecutive days three knee arthroplasties were performed, two of them done by a surgeon expert in computer assisted surgery. The rating is measured for the pre-operative, surgical and hospitalization.

**Results** There were no clinical tests requiring different equipment than the standard procedure. Informed consent to the intervention was adapted without having developed specific questions by patients. Surgery lasted for a significantly longer time than the standard procedure, and this not only for the surgeon less confident, but also for the experienced surgeon. The tibial bone cut was repeated in all 3 cases. The preparation of the femur underwent a full reassessment in one case, the distal cut in one case and the change of the size in a third case. In one case the joint balance was achieved with a 16 mm spacer. The choice of the size of prosthetic components deviated from the pre-operative planning in two out of three cases. At the postoperative radiographic alignment of the components on standard anteroposterior X-rays, each measurement was +4°, −3°, +7° for the tibia, and 0°, 3°, 0° for the femur. The post-operative blood loss and functional recovery showed no differences in the standard procedure. In one case, there was a diffuse hematoma of the anterior-medial region of the middle third of the leg. The surgical wounds healed by primary intention, the length of stay was 5, 7 and 8 days respectively.

**Discussion** The clinical course, radiographic results, surgical procedure, implant placement, and the cost of the procedure were the "audit" that stopped, in our hospital, the process for amending the procedure to the computer assisted surgery.

### Unicompartmental knee arthroplasty (UKA) revision using computer assisted technique

A. Todesca*, J. Bejui Hugues, G. Macali

Istituto Chirurgico Ortopedico e Traumatologico I.C.O.T. (Latina, IT)

**Introduction** Uni-knee represents a fascinating solution for medial or lateral knee osteoarthritis (OA) also in young people; nevertheless early aseptic loosening is not rare. When a UKA requires revision, the best outcome is achieved when it is converted to a primary total knee arthroplasty (TKA). Particularly challenging at the revision surgery is to achieve a correct lower limb alignment, a correct rotation of the distal femur with a good patellar tracking and a healthy bone support for the implants. Goal of this study was to evaluate the usefulness of computer assisted surgery (CAS) in UKA revision.

**Materials and methods** From 2008 to 2010, 8 consecutive knees with a failed UKA underwent conversion to TKA. All patients were females with the mean age of 64 years (range, 52–73). The most common failure was the tibia plateau aseptic loosening (5 patients), in one case was a secondary post-traumatic instability 4 years after the implantation, while in the last two cases there were an OA progression in the lateral compartment of the knee. All UKA were converted to a primary unconstrained, posterior cruciate ligament sacrificing, TKA using the Amplivision computer navigation system (Amplitude). We have performed the revision surgery following the same steps as in a primary implantation with a procedure based on a bone morphing acquisition performed on the surface of the original implants, followed by a dependant bone cut sequence. The axial and rotational alignment as well as the ligamentous balancing were thus planned without any influence of the bone loss determined by components removal.

**Results** In all patients failed UKA were converted in primary TKA nevertheless a metal wedge augmentation and a tibial stem were required only in one knee. All patients were followed for an average of 14.5 months. Full weight bearing X-rays exams were performed to evaluate lower-limb alignment. The mean Knee Society knee scores and functional scores at latest follow-up were 93 and 78 respectively.

**Discussion** Conversion of a failed UKA to a TKA is technically demanding, but may be done successfully with CAS technique. Use of the navigation system in UKA revision surgery allows for precise alignment of components, balance of the gaps, and filling of the bony defects by facilitating selection of appropriate implant sizes and wedges.

**Conclusions** Computer assisted revision technique allows the conversion of a failed UKA into a TKA.

### *C46—KNEE 4*

### Unicompartmental knee replacement implant survivorship with All-Poly tibial component

D. Bruni*, F. Iacono, M. Lo Presti, G. Raspugli, S. Zaffagnini, M. Marcacci

Istituto Ortopedico Rizzoli (Bologna, IT)

**Introduction** Some papers recently reported high failure rates for All-Poly tibial UKRs, indicating aseptic loosening of the femoral component and medial tibial collapse as the predominant failure mechanisms. The purpose of this study was to determine the 10-years implant survivorship and the failure mechanisms of an All-Poly tibial UKR in a large series of consecutive patients.

**Materials and methods** A retrospective evaluation of 273 patients at 3–10 years of follow-up was performed. Clinical evaluation was based on KSS and WOMAC scores. Subjective evaluation was based on a VAS for pain self-assessment. Radiographic evaluation was performed by 3 independent observers. A Kaplan–Meier survival analysis was performed assuming revision for any reason as primary endpoint. Reason for revision was determined basing on clinical and radiographic data.

**Results** The 10-years implant survivorship was 90.5%. The prosthesis was revised in 22 patients (8%) and aseptic loosening of the tibial component was the most common failure mode (11 cases). Revision was performed with an unconstrained implant in 18 cases. Mean post-operative results for KSS and WOMAC score were 87.0 and 87.37, respectively. VAS showed a significative improvement ($p < 0.0001$) respect to pre-operative condition.

**Discussion** Unlike some recent reports, this study demonstrated a satisfactory 10-years implant survivorship using an All-Poly tibial UKR. Aseptic loosening of the tibial component was the most common failure mechanism.

**Conclusions** UKR with All-Poly tibial component is a reliable option for medial unicompartmental osteoarthritis.

### Partial knee arthroplasty: our experience with PF implants and combined prostheses

S.M.P. Rossi*, L. Ghidoni, L. Perticarini, M. Ghiara, L. Piovani, F. Benazzo

Clinica Ortopedica e Traumatologica, Fondazione IRCCS Policlinico San Matteo (Pavia, IT)

**Introduction** Unicompartmental knee arthroplasty represents a safe solution and its results can be reproduced in knee surgery. Since years in addition to the replacement of tibio-femoral compartment, the possibility of the only femoral-patellar replacement is available. This approach has produced variable outcomes in the literature.
**Materials and methods** From 2008 to 2010 in our Institution 45 patients underwent femoral-patellar joint replacement with new generation implants (Journey PFJ Smith and Nephew and PFJ Zimmer, Warsaw). In 20 patients the isolated femoral-patellar implant was performed, whereas the other 25 patients underwent the femoral-patellar replacement combined with an unicompartmental implant that was medial in 23patients and lateral in 2 cases. The unicompartmental implants used were Accuris (Smith and Nephew) or ZUK (Zimmer, Warsaw). 37 patients were women and 8 patients were men. The patients' mean age was 66.5 years at the time of operation, with a mean follow-up of 20 months (range, 6–36 months). The clinical and radiological data were analyzed using the HSS and KSS scores at 1–3–6–12 month of the follow-up and than yearly.
**Results** Two implants required revision: one implant of the femoral-patellar replacement group underwent patellar revision, whereas one implant of the combined implant group failed and underwent revision cause infection. The clinical outcomes evidenced a final mean HSS score of 97.1 and a mean KSS score of 97.2 (knee) and 96.8 (functional). There were no cases of radiologically aseptic mobilization.
**Discussion** Unicompartmental knee arthroplasty represents an innovative and effective solution for bone tissue sparing surgery and to preserve the proprioceptive system and the cruciate ligaments. On the other side these replacements, to obtain a good and reproducible outcome, need an exact patient selection with strict indications respect, and an accurate surgical technique from a well trained surgeon on unicompartmental knee arthroplasty.
**Conclusions** Our experience show good short-term results both with femoral-patellar isolated replacement and combined technique. A longer follow-up is needed for long-term results.

### Unicompartmental knee prostheses: comparison between tibial All-Poly and metal-back. Personal experience

F. Rodolfo Masera*

Città Studi (Milan, IT)

**Introduction** We show a retrospective study on unicompartmental knee prostheses. 2 groups: same femoral component, 2 tibial components, a monolithic one All-Poly and a modular one composed by metal-back and PE liner. Purpose of this study is the comparison of results obtained in the 2 groups, assuming that the different elasticity of the 2 systems can differently effect the reaction on the bone.
**Materials and methods** 100 of ZUK unicompartmental implants 2004–2007 were considered. Case histories: 65% women; average age 68 years; 95% medial and 5% lateral pathology; 70% of them had already undergone an arthroscopy. *Surgery technique*: minimidvastus access, no tourniquet; average duration of the surgery: 79 min. In 30% of the cases an associated diagnostic arthroscopy was made before implantation, to exclude arthrosis of the external and of the patellofemoral compartment and to treat possible lateral meniscus pathologies. Rehabilitation *Protocol*: same. Same femoral component with anatomic design was used. The tibial plate is available in two different configurations: Unicompartmental All-Poly (50% 2004–2005) or modular, composed by metal-back and PE liner (remaining 50%). The choice of the implant has knowingly not been made on basis of age, sex, deformation or others. *Preparatory Evaluation*: always 2 X-rays projections with knee joint under load, Rosenberg and axial patella; often standard RMN. Post-operative Evaluation: at 1 month, both clinic and X-rays, clinic only at 3 and 6 months, then annual X-rays. *Latest evaluation*: beginning of 2011; follow.up, 3–6 years.
**Results** Having evaluated the post-operative recovery, the mobility and the vanishing of the pain, the clinic results are superimposable. Also the radiographic data evaluated in a long period did not show any difference in terms of keeping the correct position of the implant in place. We also noticed by X-rays occasional lucencies and sclerosis of the tibial plate underneath the implant, equal distributed in the two groups. *Revisions*: Metal-back: 1 due to early surface infection; 1 to subsequent ACL traumatic injury; 1 to progression of the arthrosis of the femur-patella and of the lateral compartment. All-Poly: 1 due to early sinking of the tibial component and subsequent total replacement, 1 to progression of the arthrosis.
**Conclusions** There was no difference in the surviving on the basis of our tests and we cannot give any indication on the choice of one or of the other prosthesis. Different indications could only be given after a longer follow-up.

### Survivorship of unicompartmental knee replacements in spontaneous osteonecrosis

D. Bruni*, F. Iacono[1], M. Lo Presti[1], S. Zaffagnini[1], M. Marcacci[1]

Istituto Ortopedico Rizzoli (Bologna, IT)

**Introduction** Reports regarding implant survivorship in unicompartmental knee replacements (UKRs) performed for spontaneous osteonecrosis of the knee (SPONK) often derive from small series with an average follow-up of 5 years, enabling to generate meaningful conclusions.The purpose of this study is to determine the 10-year survivorship of the implant in a large series of patients with SPONK of the medial compartment treated with a UKR.
**Materials and methods** A retrospective evaluation of 84 consecutive patients with late stage SPONK at a mean follow-up of 98 months was conducted. All patients received a pre-operative MRI to confirm the diagnosis, to exclude any metaphyseal involvement and to assess the absence of significative degenerative changes in the lateral and patella-femoral (PF) compartment. Mean age at surgery was 66 years and mean body mass index (BMI) was 28.9. In all cases SPONK involved the medial compartment. A Kaplan–Meier survival analysis was conducted assuming revision for any reason as endpoint. Reason for revision was detected basing on radiological and clinical evaluation.
**Results** The 10-year Kaplan–Meier survivorship was 89%. Ten revisions were performed and the most common reasons for revision were subsidence of the tibial component (4 cases) and aseptic loosening of the tibial component (3 cases). No patient underwent revision for osteoarthritis progression in the lateral or in the patello-femoral compartment.
**Discussion** The presented results suggest that medial UKR performed for late stage SPONK of the medial tibio-femoral compartment in selected patients provides a 10-years Kaplan–Meier survivorship of the implant of 89%. Subsidence of the tibial component and aseptic loosening of the tibial component are the most common reasons for revision.
**Conclusions** UKR is a valid treatment option for medial compartment spontaneous osteonecrosis.

### Mid-term results in total knee revision surgery

M. Schiraldi*, G. Stennardo, G. Bonzanini, V. De Tullio

Ospedale Santi Antonio e Biagio, ASO AL (Alessandria, IT)

**Introduction** The aim of this study was to report the results of the total knee revisions surgery and the influencing factors of the outcome.

**Materials and methods** Since May 1999 to May 2007, 96 total knee revisions (TKR) were performed by the same Author (M.S.) in 94 patients. The patients were 31 men and 63 women, the mean age was 68 years (range, 56–84 years) at the time of the revision. Patients were divided into four groups: Aseptic loosening, septic loosening, active infection, components malpositioning.

**Results** The eighty-seven percent of the patients had a significant pain relief with total or partial satisfaction. The active range of motion improved significantly at follow-up. In a case the revision group in 2 steps for septic loosening was required subsequent amputation. Two cases required further revision. The average follow-up was 5.7 years (maximum follow-up 11.5 years, minimum 3.5 years). The Knee Society score was an average of 33 points before the revision and improved to 78 at final follow-up.

**Discussion** The results in revision knee surgery should be evaluated usually as clinical and functional improvement, because is unrealistic to expect an absolute improvement in a surgery that, in some cases, takes on the characteristics of a real limb salvage surgery. We found a significant difference of the results in relation to etiology: the aseptic loosening group gave the best results.

**Conclusions** The bone loss is a major concern in total knee revisions and should be classified (classification A.O.R.I. Anderson) in order to choose the best surgical strategy (use of cement, augments, morcellized bone grafts, structural grafts, prosthetic intramedullary rods) with the aim to restore the ideal joint line, get a good biomechanics of the prosthetic joints and provide stability to the system.

### *C47—KNEE 5*

### NexGen LPS total knee arthroplasty: 10-year follow-up

A. Bistolfi*, G. Massazza, F. Rosso, F. Lagalla, V. Gaito, D. Deledda, M. Crova

Dipartimento di Ortopedia e Traumatologia e MdL, AO CTO/M. Adelaide, Università degli Studi di Torino (Turin, IT)

**Introduction** Total knee arthroplasty (TKA) offers excellent results and survival in the long term. The rotating platform was developed as an evolution of the fixed one to allow a more physiological movement and to reduce the stresses on the polyethylene. This study is a prospective evaluation of 332 TKA NexGen® (Zimmer, USA) rotating platform implants.

**Materials and methods** Between 2000 and 2005, 332 TKA were performed (249 patients, 83 bilateral) in 263 women and 69 men (mean age, 71.2 years). The indications to the surgery were: 318 (95.8%) knee arthritis, 10 (3.1%), rheumatoid arthritis, 4 (1.1%) unicompartmental prosthesis loosening. The patellar replacement was performed in 162 cases (48.8%). For the clinical evaluation HSS questionnaire was used, and for the radiographic one the "Knee-Society-Roentgenographic-Evaluation-System" Data were prospectively collected on a database specifically developed.

**Results** 229 patients (91.8%, 305 cases) are completed the study, with average follow-up of 77.4 months (SD, ± 18.4 months). The range of motion increased from 66.9° (SD, ± 9.6°) to 113.5° (SD, ± 18.8°) and the total score from 54.6 points (SD, ± 7.2) to 86.3 (SD, ± 6.4; $p < 0.05$). 5 cases (2.1%) underwent to revision surgery (1 arthrodesis, 4 implant revision); the cumulative survival rate was 98.4% (SD, ± 0.8%). In 90 cases (39.3%) radiolucent lines were found, but only in 5 cases (2.1%) were progressive. The average angles showed good alignment.

**Discussion** Fixed platform total knee arthroplasties are the gold-standard, while some authors suggested doubts regarding the rotating implants, for both the utility that this type of implant might have and their resistance to stress and wear of the rotating polyethilenic surface. No failures because of polyethylene rupture, or because of tibial osteolysis for increased abrasion of the insert were detected. The functionality and the range of motion were completely recovered. With this prosthesis good clinical and radiographic results were obtained, with a cumulative survival amounted to 98.4%. The results support the use of the rotating platform, even though the data do not allow a comparison with the NexGen fixed platform model.

**Conclusions** The NexGen® prosthesis provides good results in the medium to long term, particularly with the use of cementation and rotating tibial platform.

### Our experience with the LCCK implant in knee arthroplasty

S.M.P. Rossi*, L. Piovani, L. Perticarini, A. Colombelli, F. Benazzo

Clinica Ortopedica e Traumatologica, Fondazione IRCCS Policlinico San Matteo (Pavia, IT)

**Introduction** LCCK implant (Zimmer, Warsaw) represents an important solution both for knee prostheses revision, and for the first implant cases that need higher constraint, as in articular and extra articular major deformities situation, infection outcomes, important instability.

**Materials and methods** From 2004 to 2010 in our Institution 125 LCCK prostheses were implanted in119 patients (3 bilateral). In 60 cases the implant was used as revision and in 65 as first implant. The main diagnosis was osteoarthritis both in revisions (first diagnosis) and primary implant cases (93%). The main revision cause was aseptic loosening for malpositioning or malalignment (55%). The patients were followed-up at 1–3–6–12 months and then yearly using HSS and KSS scores for the clinical evaluation and using KSS score for the radiological data.

**Results** The mean HSS and KSS (knee and functional scales) scores raised from 33, 38 and 31 before surgery to 88, 92 and 85 at the and of the follow-up. There was 10% of complications in the revisions and 5% in the first implant group. The Kaplan–Meier survival rate was 93% (95% confidence interval, 94% to 100%). The mean radiological ß angle was 89.6° (89–92), the mean s was 84.2° (82–87) for the tibial side. Concerning the femoral component the mean angle was 94.8° (92–96) and the mean ? was 5.1°(4–7). There were no modifications of these measurements or clinical and radiological signs of mobilization during the follow-up.

**Discussion** The main goal in the first implant surgery and also in the revisions is good clinical and radiological outcomes that preserve distance with low percentage of failure.

**Conclusions** In our experience the use of the LCCK implant was reliable and showed reproducible results with a complications percentage of 10% in the revisions and of 4% in the primary implants. Our results need a longer follow-up, but they are well comparable with the data in the literature with other comparable implants.

### Trabecular metal cones for severe bone defect in knee revision arthroplasty

D. Tigani*[1], M. Fosco[2], E. Crainz[1], G. Vox[1]

[1]U.O. Ortopedia e Traumatologia, Policlinico Santa Maria alle Scotte (Siena, IT);
[2]Istituto Ortopedico Rizzoli (Bologna, IT)

**Introduction** Porous tantalum is a transition metal available to be used in orthopaedic surgery in its trabecular form (TMT). Porous tantalum has an excellent biocompatibility and a high resistance and osteoinductive characteristics. Tantalum-made augments are largely used in knee prosthetic surgery: augments in a block and wedge fashion are useful in managing small amounts of bone loss; nevertheless to compensate for larger bone defect involving metaphyseal segment or major portion of the condyle or plateau, tantalum cones have been recently developed.
**Materials and methods** We present our experience with femoral and tibial tantalum cones in 11 knees (12 cones overall were used, six on femur and six on tibia) during knee revision arthroplasty. Both cemented and cementless cones were used. Patients were assessed clinically using American Knee Society score (AKSS) and radiographically (with American Knee Society radiographic evaluation system), before surgery and at every postoperative control.
**Results** After a mean 30 months follow-up (range, 12–56 months) there were no radiological signs of aseptic loosening or migration of the components; TMT cones showed good osteointegration. The only reported post-operative complication was delayed union of the tibial tuberosity in a patient who required osteotomy for surgical exposure. All patients improved according to AKKS, both clinically (from 42 points mean to 69 points mean after intervention) and functionally (from 14 points mean to 69 points mean after intervention).
**Discussion** Although early experiences with TMT tibial cones have been recently published, our series represents the first experience with cones in both femur and tibia. Our results supports use of porous tantalum metaphyseal cones as a viable option for revision knee arthroplasty with large amount of bone defects in both femur and tibia. Compared with the preparation of a structural bone allograft, TMT metaphyseal cones have several advantages: simpler surgical technique of implantation, resulting perhaps in shorter operative times with a potential benefit of a decreased infection risk; the risk of disease transmission is minimized. Nevertheless TMT cones have increased cost. We think, based to recent experiences, there are no particular difficulties in removing these components.
**Conclusions** TMT cones represent an effective alternative to structural bone allograft to compensate for large bone defect during revision knee arthroplasty. Compared with these, TMT cones have simpler surgical technique, providing optimal mechanical support and rapid osteointegration.

### Biomechanics of knee prosthesis: study of periprosthetic bone remodelling by DXA

M. Arduini*, I. Cerocchi, L. Saturnino, A. Scialdoni, F.L. Perrone, U. Tarantino

UOC Ortopedia e Traumatologia, Fondazione Policlinico Tor Vergata, Università degli Studi Tor Vergata (Rome, IT)

**Introduction** Bone tissue at the proximal tibia provides mechanical support to the tibial component in Total Knee Arthroplasty (TKA). Bone strength is critical to the anchorage of the tibial component. Bone quality varies with age and sex, articular condition, level of physical activity and it is influenced by the metabolic state of the skeleton. Aim of the study was to analyze the influence of pre-operative bone mineral density (BMD) at the proximal tibia, evaluated by DXA, on periprosthetic bone remodelling.
**Materials and methods** Thirty patients were included in the study (average age, 65 years; range, 55–75), 18 females and 12 males, who underwent MD measurement at the lumbar spine (L1-L4), at the proximal non-dominant femur and at the proximal tibia before intervention. BMD evaluation at the proximal tibia was repeated at 3, 6 and 12 months after implantation of a cemented knee prosthesis. Both pre-operatively and at the follow-up visits ROM and Oxford Knee Score were evaluated. Osseointegration and eventual migration of the tibial component were identified by X-rays.
**Results** Pre-operative DXA allowed the distinction between osteoporotic ($n = 6$), osteopenic ($n = 15$) and normal ($n = 9$) subjects. BMD values at the proximal tibia after intervention documented a decrease in BMD at ROIs 1, 2 and 3 in all patients. This phenomenon proved to be particularly evident in individuals with osteoporosis or severe osteopenia. Bone remodelling was slower and less evident at ROI 3 (central) in all cases. BMD decrease at ROI 1 (medial) and 2 (lateral) was related to pre-operative load axis of the knee (varus or valgus).
**Discussion** Average BMD reflected patient's bone quality. BMD distribution in the different parts of the tibia was influenced by pre-operative alignment of the knee. Even after the load axis has been corrected the tibial component lies on a surface in which BMD distribution is asymmetrical. On the less loaded surface, BMD rapidly increases after axis correction, but this is less evident in osteoporotic subjects.
**Conclusions** Pre-operative bone quality influences osseointegration of the tibial component, and restoration of a correct axis causes BMD re-distribution, thus increasing implant stability.

### Use of Tantalum tibial plateau in uncemented total knee arthroplasty

G. Cattaneo*, L. Romano, S. Tornago, A. Camera

Ospedale "Santa Corona" (Pietra Ligure-Savona, IT)

**Introduction** The aim of this study was to investigate, with preliminary clinical and radiographics results, the use of Tantalum trabecular metal in uncemented primary TKA for a biological fixation of tibial plateau.
**Materials and methods** We performed 51 of tka in the period between 08/03/2007 and 22/10/2009 with the system Nexgen Lps (Zimmer®) using Tantalum uncemented tibial plateau.
**Results** The mean age was 63 (max. 78, min. 35), the mean follow-up was 6 months (max. 2.5 years, min. 2 months). Preoperative and Postoperative clinical and radiographical evaluation were collected using telemetry of inferior limb and X-rays in maximal flexion and full extension. The evaluation of clinical outcome was made using Tegner-Lysholm Knee score.
**Discussion** We observed a high functional outcome in 95% percent of patients as well as a good bone integration in 3 months of the tibial pegs on the tibial bone, showed in the radiographics controls. No aseptic loosening was documented on follow-up, no early mobilitation occured in our study. We have one patient with tardive infection of the implant.

**Conclusions** Clinical and radiographics results and patients satisfacion suggest that the way we have to follow is the use of uncemented Tantalum plateau in TKA especially in patients <60 years.

### CT evaluation of polyethylene wear in total knee arthroplasty with a ceramic femoral component

M. Manili*, F. Pezzillo, P. Colletti, C. Barresi, R. Giacomi

San Carlo-IDI (Rome, IT)

**Introduction** Polyethylene wear, one of the main causes of knee arthroplasty failure, depends on the quality and physical characteristics of the coupling material. The increasing demand for total knee arthroplasty (TKA) in younger and more active patients has pushed research towards the development of innovative materials (such as ceramic) to couple to polyethylene. The aim of this study is to evaluate polyethylene wear in TKA with a BIOLOX® delta ceramic femoral component through computerised axial tomography (CT).
**Materials and methods** Between 2007 and 2010, 10 patients had TKA with a cemented BIOLOX® delta femoral component, a cemented Ti6aI4 V tibial plate with EtO sterilised polyethylene liner. The patients were assessed preoperatively and at 3, 6, 12, 18 months with Hospital for Special Surgery score (HSS score), ROM and standard X-rays. Multislice CT scans were performed at 3, 6, 12 months to evaluate the presence of scratches or gaps in the polyethylene.
**Results** The average follow-up was 12 months. All patients reported an increase of the HSS score (from 43.3 to 86.2 at the last follow-up) and ROM (from 97° to 123°). No radiolucent lines were found at the ceramic-cement and cement-bone interfaces. All CT sequences highlighted the absence of scratches or gaps on the liners articulating surface, which can be associated to wear. No implant-related complications and no revision occurred.
**Discussion** The CT analysis is useful for evaluating the rotation of femoral and tibial components, for studying the bone-cement interface and for evaluating wear and possible polyethylene gaps. The presence of the ceramic femoral component reduces metal artefacts upon execution of the CT and therefore it is possible to show polyethylene gaps up to 600 microns in size. Thanks to standardised repetition of the CT analysis using the same equipment, orientation and distance during image acquisition, it is possible to monitor the evolution of polyethylene wear over time.
**Conclusions** Radiographic and CT results, even though at a short follow-up, demonstrate that the Multigen Plus Ceramic Knee prosthesis constitutes an excellent solution to problems of wear.

### Ganglion cyst as a cause of peroneal nerve palsy: etiopathogenesis and surgical treatment

G. Logroscino*, V. Ciriello, G. Malerba, E.D. Pagano, C. Fabbriciani

Istituto di Clinica Ortopedica, Università Cattolica del Sacro Cuore (Rome, IT)

**Introduction** Intraneural ganglion cyst of the peroneal nerve (PN) is a rare disease and the pathogenesis is not well defined and many different and conflicting etiological hypotheses have been proposed. PN ganglion cysts have some particular features, such as the proximity of the cyst to the proximal tibio-peroneal joint (PTFJ), the preferential involvement of the deep branch of the peroneal nerve (DPN), the rare isolated involvement of the superficial branch of the peroneal nerve (SPN) and the high percentage of recurrence (10–20%). The aim of this study is to report a case of intraneural ganglion cyst of the peroneal nerve as a cause of unexplained peroneal paralysis.
**Materials and methods** The case report of a 65 years old female who initially referred pain in the right knee, associated with a progressive motor deficit of the ankle and foot dorsiflexion. The neurological impairment evolved from a weakness of the extensor communis digitorum (EDC) and numbness of the first dorsal interdigital space (DPN) to the final complete paralysis. An ultrasound scan showed an hypoechogen cystic formation next to the peroneal nerve close to the head of the fibula with a connection to the PTFJ space. The electromyography examination (EMG) showed an axonal involvement of the PN. The MRI showed the typical "tail sign". Surgical exploration of the PN revealed a cystic dilatation of the articular branch (AB) of the PN with involvement of the DPN. The resection and ligation of the AB of the PN and decompression of the cyst with resection of the upper PTFJ was surgically performed.
**Results** The patient showed a steady clinical and elecromyographic improvement after the surgical procedure. EMG analysis at 16 months showed no signs of denervation, clinical examination showed F5 for tibialis anterior muscle, F5 for EDC and F3 for extensor allucis longus muscle.
**Discussion** The intraoperative findings and the functional recovery confirmed the "unifying theory" (Spinner et al.), whereby the disease arises from a primary pathology of PTFJ (repetitive microtrauma). The overproduction of synovial fluid originates from a primitive degeneration of the PTFJ using the AB as a conduit through a valve mechanism, the intracapsular fluid would lead to the formation of a perineural cysts and peripheral nerve compression.
**Conclusions** Surgical treatment with ligation and section of the AB branch and resection of the PTFJ showed to be successful in order to stop the disease progression and reduce the risk of relapse, with complete clinical recovery.

### Accuracy of standard instruments in performing tibial resection in TKA: a case study

R. Iorio*, G. Bolle, F. Conteduca, L. Valeo, D. Mazza, A. Ferretti

Ospedale Sant'Andrea (Rome, IT)

**Introduction** The aim of this study was to verify the accuracy of conventional instruments in the tibial resection of total knee arthroplasty using navigation system, to obtain an estimation of the error in the manual positioning of the tibial guide, the error in the plan of resection and of the deviation due to the impact of prosthetic components.
**Materials and methods** Forty primary total knee arthroplasties were performed by the same surgeon. The resection guide was first positioned with conventional instruments and before executing the osteotomy, the accuracy of the guide positioning was evaluated via the navigator. The alignment measure was repeated after resection effectuated following navigator's data, evaluating the difference between values suggested by navigator and those obtained after the resection, and after component implantation, thus quantifying the deviation due to the positioning of components. Results deviations = 1.5° have been considered satisfactory.
**Results** Coronal plane unsatisfactory results were 15% in manual positioning of the resection guide and 10% after effective resection, showing in both cases a high prevalence of varus deviation (62.5% and 45%). A deviation of >3° has never been found. On the sagittal plane, the unsatisfactory results were respectively 45% in manual positioning of the resection guide and 40% after effective resection, showing in both cases, a prevalence of values sloop smaller than ideal (60% and 80%). The deviations due to the procedure for implantation of the tibial component were never greater than 1.5° both on the sagittal and coronal plane.

**Discussion** This study showed that it is more difficult to obtain proper alignment on the sagittal plane than on the frontal plane. Although by statistical analysis significant errors were not detected, with the exception of rare cases, it is possible to envisage that in an elevated percentage of cases errors may potentially add up events of major importance, considering the prevalence of deviations in varus and with loss sloop degrees. The procedure for the introduction of the tibial component does not seem to substantially change the orientation.

**Conclusions** The advantage of computer-assisted technique is the possibility to check every single step of the intervention and to perform continuously even small corrections, limiting the possibility of significant errors due to the sum of minimum deviations.

### *C48—KNEE 6*

### High tibial osteotomy (HTO) for knee arthritis treatment: mid-term clinical and X-ray evaluation

M.F. Surace*[1], E. Bulgheroni[1], P. Bulgheroni[2], D. Marcolli[1], A. Fagetti[1], P. Cherubino[1]

[1]Università dell'Insubria (Varese, IT);
[2]Ospedale di Circolo (Varese, IT)

**Introduction** The high tibial osteotomy is a procedure frequently used for the treatment of medial arthritis of the knee due to a lower limb mallalignement. Aim of this procedure is to ease pain, improve knee function and slow down the arthritis progression and eventually delay the knee arthroplasty through the correction of the lower limb-bearing axis. The results reported in literature vary considerably, but generally this procedure seems to grant a good pain reduction and a knee function improvement in the 80–90% of patients with a 5 year follow-up and in the 50–65% at 10 years of follow-up.

**Materials and methods** We have evaluated the clinical and X-ray results at a mean follow-up of 5 years of 42 patients that were treated with a closing wedge high tibial osteotomy. The data collected through the clinical evaluation scales (IKDC, Lysholm tegner and Knee rating scale) during the follow up and through the preoperative and follow-up X-rays were intended to verify the stability of the achieved results and to prove the possible relationship between the anthropometric parameters with the activity performed or the arthritis. The statistical analysis was performed through the SPSS 11.0 software.

**Results** The 92% of patients during the follow-up did not need a knee arthroplasty. The clinical evaluations performed preoperatively and during follow up showed a statistically significative improvement of the subjective evaluation pain scores (IKDC) and a fair improvement of the evaluation scales during follow up in relationship with age, and in particular for the Tegner activity scale. The mean correction angle was of 8°, with a higher angle of correction for older patients. It has been reported only a slight loss of correction after 5 years, with a decrease of the functional results. In the females the correction performed was lower, but the loss of correction was higher.

**Discussion:** In the present study we have emphasised how the high tibial osteotomy is able to grant satisfaction for the patient and a good level of activity in selected individuals in certain circumstances. The clinical and X-ray results are considered good at a medium term follow up even if it has been noted a slight arthritis progression with aging. Age, BMI and the entity of the correction loss are factors that seem to be connected to the good objective and subjective outcome after surgery.

**Conclusions** HTO delivers, in selected patients, optimal subjective satisfaction and functional rates.

### Proximal high tibial osteotomy for osteoarthritis of the knee

F. Specchiulli*, M. Rendina, M. Berardi

Cattedra di Malattie dell'Apparato Locomotore, Università degli Studi di Foggia (Foggia, IT)

**Introduction** The aim of HTO is to unload the medial compartment by changing the axial alignment and correct load imbalance.

**Materials and methods** We carried out a retrospective study of 93 patients who underwent closed wedge high tibial osteotomy for severe medial compartment osteoarthritis between 1987 and 1997. A total of 60 patients (65 knees) were available for review at a mean of 15 years (10–20). Weber's procedure was used in all of the osteotomies. Clinical evaluation was done with the knee society clinical rating system.

**Results** Kaplan–Meier survival was 95% at 5 years, 75% at 10 years, 58% at 15 years. Twenty-two knees required conversion to a TKR at a mean time of 12.5 years.

**Discussion** Lateral closing-wedge HTO must be considered a difficult procedure with an unpredictable outcome. Its disadvantages include the risk of damage to the common peroneal nerve and the need for fibular osteotomy or disruption of the proximal tibiofibular joint. All the studies confirm that the "time" influences the long term stability of the results. More than 50% of our knees showed worsening of knee arthritis. The Weber's procedure has undeniable advantages over traditional and new methods of fixation. This technique allows immediate weight-bearing and early knee motion and ensures the maintenance of correction until consolidation of the osteotomy.

**Conclusions** Several factors may affect the post-operatively result of a high tibial osteotomy, including under or overcorrection, severe varus malalignment, fixation failure, preoperative range of motion and flexion contracture. Careful patient selection and precise correction of the deformity are the main factors of success.

### Necrosis of the femoral condyle: mini-invasive treatment with the use of biotechnology

G.M. Calori*, M. Bucci, P. Fadigati, E. Mazza, M. Colombo, C. Ripamonti

C.O.R., Istituto Ortopedico G. Pini (Milan, IT)

**Introduction** Osteonecrosis of the femoral condyle is a known cause of spontaneous onset of pain, acute and painful, usually borne by the anterior-medial side of the knee joint. Treatment is conservative in the initial states of disease. The surgical procedures include arthroscopic debridement, osteotomy, drilling of the lesion with or without bone grafting, allograft and prosthetic replacement.

**Materials and methods** Five patients diagnosed with osteonecrosis of the medial condyle were treated at our facility with core decompression and local application of growth factors (rhBMP-7). Patients were evaluated at 3, 6, 12 and 24 months by physical examination quantified by Knee Society knee scores and X-rays.

**Results** Four of five operated knees were treated successfully without radiographic progression of disease or use of prosthetic replacement. Two-year follow-up four patients had pain relief and absence of radiographic progression of the disease. A patient was considered as failed presented as a complication of heterotopic ossification at the level of the vastus lateralis quadriceps that has influenced clinical results.

**Discussion** The proposed technique seems to be a successful attempt to save the femoral condyles with osteonecrosis, it also not preclude subsequent treatment such as prosthetic replacement. The failure observed in our trial let us underline the importance of sealing the "biological room" created to the application of growth factors and highlights the ability of these biotechnologies in the process of bone regeneration.

**Conclusions** We consider this method an effective alternative for the treatment of osteonecrosis of the femoral condyle in stage IV, where the gold standard remains, without doubt, prosthetic replacement.

### Knee arthrodesis with a press-fit, modular intramedullary nail without bone-on-bone fusion after infected revision TKA

F. Iacono*, M. Lo Presti, D. Bruni, G. Raspugli, M.P. Neri, A. Bondi, M. Marcacci

Istituto Ortopedico Rizzoli (Bologna, IT)

**Introduction** Knee arthrodesis can be an effective treatment after an infected revision Total Knee Arthroplasty (TKA). The main hypothesis of this study is that a two-stage arthrodesis of the knee using a press-fit, modular intramedullary nail and antibiotic loaded cement, to fill the residual gap between the bone surfaces, prevents an excessive limb shortening, providing satisfactory clinical and functional results even without direct bone-on-bone fusion.

**Materials and methods** The study included 22 patients who underwent knee arthrodesis between 2004 and 2009 because of recurrent infection following revision-TKA. Clinical and functional evaluations were performed using the Visual Analogue Scale (VAS) and the Lequesne Algofunctional Score. A postoperative clinical and radiographical evaluation of the residual limb-length discrepancy was conducted by three independent observers.

**Results** VAS and LAS results showed a significant improvement with respect to the preoperative condition. The mean leg length discrepancy was less than 1 cm. There were two recurrent infections that needed further surgical treatment. We had two recurrent infections, that needed a new surgical revision.

**Discussion** This study demonstrated that reinfection after R-TKA can be effectively treated with arthrodesis using a modular intramedullary nail, along with an antibiotic loaded cement spacer and that satisfactory results can be obtained without direct bone-on-bone fusion.

**Conclusions** The two-stage arthrodesis with the use of intramedullary nail, provides a painless, stable and functional limb. The rate of recurrence of infection of 14.28%, remains a challenging problem and it has to be kept in mind both of the surgeon and the patient when knee arthrodesis is considered after infected revision-TKA. A failure of arthrodesis for this reason frequently requires.

**Suggested readings**

1. Lequesne M, Mery C, Samson M, Gerard P (1995) Indexes of severity for osteoarthritis of the hip and knee. J Rheumatol 65:85–89
2. Rand JA (1993) Alternatives to reimplantation for salvage of the total knee arthroplasty complicated by infection. J Bone Joint Surg 75-A:282–289
3. Hanssen AD, Trousdale RT, Osmon DR (1995) Patient outcome with reinfection following reimplantation for the infected total knee arthroplasty. Clin Orthop 321:55–67

### Lipoma arborescens of the knee: surgical treatment

D. Mascello*, M. Mariani, O. Mazza, M. Crostelli

Rome, IT

**Introduction** Lipoma arborescens is a not very frequent intra-articular benign tumour most commonly found in the knee. Extensive or diffuse synovial proliferation of fatty tissue characterizes the disorder. Etiology is unknown. Patients with lipoma arborescens of the knee present with pain, with no or moderate effusion.

**Materials and methods** We report four cases of lipoma arborescens of the knee. Three patients were males and one female; mean age, 47 years; age range, 23–68 years. No history of trauma was present. Magnetic resonance imaging (MRI) was performed in every patient. Patients were treated surgically. Three of the patients were treated arthroscopically and the other one with arthroscopic and open procedure. The median follow-up was 31 months (range 24–47 months). In two of the four patients (23 and 42 years) the lipoma arborescens was localized in the suprapatellar pouch and was removed by arthroscopic synovectomy. In the third patient (55 years) the lipoma arborescens was located in the suprapatellar pouch, in the intercondylar notch and between the ACL and PCL. In this patient was present also a horizontal tear in the posterior horn of the medial meniscus. Lipoma was removed arthroscopically. In the fourth patient (68 years) MRI revealed the presence of the lipoma arborescens in the suprapatellar pouch, in the intercondylar notch and in lateral gutters and also the coexistence of osteoarthrosis. The lipoma arborescens was resected arthroscopically with poor results. Six months later open procedure was performed.

**Results** In the first two patients excision of the lipoma led to immediate relief of the symptoms. The third patient still presents moderate effusion. After the open surgical treatment the fourth patient still complains moderate pain and effusion.

**Discussion** Lipoma arborescens is not very frequent in adulthood and it rarely occurs in childhood. It is more frequent in the fourth and fifth decade. Knee osteoarthrosis is often coexistent in the elderly. The choice regarding open versus arthroscopic synovectomy depends on location and concomitant lesions.

**Conclusions** We concluded that arthroscopic synovectomy can be used when lipoma arborescens is confined to the anterior knee compartment.

### Wound complications following total knee arthroplasty: prevention and options of treatment

A. Schiavone Panni[1], M. Vasso*[1], S. Cerciello[1], M. Salgarello[2]

[1]Dipartimento di Scienze per la Salute (Campobasso, IT);
[2]Dipartimento di Chirurgia Plastica UCSC (Rome, IT)

**Introduction** Wound complications following total knee arthroplasty can lead to complex soft tissue defects with possible exposure of bone and/or hardware, high risk of infection and loss of the prosthesis or even of the limb. The aim of our study is to present an algorithm of treatment of knee periprosthetic soft tissue defects, relative to their extent and depth. Different management of exposed total joints is also proposed, depending on the presence or less of infection and on the timing of infection itself.

**Materials and methods** In accordance with literature and our experience, it has been thoroughly analyzed the incidence and risk factors, the prevention and possible treatment options of wound complications following total knee arthroplasty.

**Results** There is much controversy regarding the optimal management in skin necrosis around a total knee. Prevention certainly represents the best management, choosing the most appropriate incision, avoiding narrow skin incisions, and being careful of excessive lateral retinacular release and early postoperative flexion. Frequent dressings, debridement, fasciocutaneous, muscle and perforator flaps have been differently used.

**Discussion** Muscle coverage remains the standard to which all other flaps should be compared, especially in cases of infected wounds. Perforator flaps have represented, in the last 15 years, a true revolution in soft tissue reconstruction around the knee. Both flaps, muscle and perforator, provide a better local circulation, so facilitating treatment of eventual deep joint infection.

**Conclusions** When wound complications occur, prompt management is mandatory. An algorithm for treatment of wound defects is presented, available for both primary and revision knee replacement.
